# Youth-led spatio-temporal heatwave co-adaptation mapping through data-driven participatory dotmocracy approach

**DOI:** 10.1007/s00484-026-03218-0

**Published:** 2026-05-08

**Authors:** Zulfaqar Sa’adi, Zainura Zainon Noor, Nur Syamimi Zaidi, Nurul Hana Mohamed, Fateha Abdul Razak, Mohd Faiz Foze, Siti Fadilla Md Noor, Md Abdullah Al Mamun Hridoy, Shamsuddin Shahid, Farnaz Ershadfath, Ricky Anak Kemarau

**Affiliations:** 1 Centre for Tropical Climate Change System, Institute of Climate Change, Universiti Kebangsaan Malaysia, Bangi, Selangor 43600 Malaysia; 2 Centre for Environmental Sustainability and Water Security (IPASA), Research Institute for Sustainable Environment (RISE), Universiti Teknologi Malaysia (UTM), 81310 UTM Johor Bahru, Johor, Malaysia; 3 Faculty of Civil Engineering, Universiti Teknologi Malaysia (UTM), Johor Bahru, Johor 81310 Malaysia; 4 Faculty of Chemical and Energy Engineering, Universiti Teknologi Malaysia (UTM), 81310 Johor Bahru, Johor, Malaysia; 5 Faculty of Fisheries, Sylhet Agricultural University, Sylhet, 3100 Bangladesh; 6 Regional Climate Change Center, National Center for Meteorology, Jeddah, 23225 Saudi Arabia; 7 Environmental and Atmospheric Sciences Research Group, Scientific Research Center, Al-Ayen University, Thi-Qar, Nasiriyah, 64001 Iraq; 8 Water Engineering Department, School of Agriculture, Shiraz University, Shiraz, Iran; 9 Earth Observation Centre, Institute of Climate Change, Universiti Kebangsaan Malaysia, Bangi, Selangor 43600 Malaysia

**Keywords:** Heatwave adaptation, Participatory mapping, Youth engagement, Co-creation, Malaysia

## Abstract

Heatwaves are increasingly impacting rural communities in Malaysia, with disproportionate effects on youth due to limited adaptive capacity and a lack of targeted interventions. This study presents a data-driven, participatory heatwave co-adaptation mapping initiative involving thirty Form Three students from a rural high school in Segamat, Johor. The structured, interactive workshop comprised five core activities: emoji-based reflection and gamification exercises to elicit emotional responses and visualize the climate change process; a Mini Talk with interactive materials and quizzes on local climate trends and adaptation options; and hands-on group dotmocracy mapping sessions to identify cold and thermal hotspots, assess vulnerabilities, and propose adaptation measures. Analysis of the school environment revealed a long-term warming trend, with daily Tmax increasing by 0.022 °C per year (≈ 0.22 °C per decade; t = 46.86, *p* < 2 × 10⁻¹⁶) from 1950 to 2022. March recorded the highest frequency of extreme heat days (> 35 °C) with 160 days (7.07% of March days), and Level 1 heatwaves (> 35 °C) occurred 46 times, lasting 3–14 days, whereas only 2 Level 2 events (> 37 °C) were observed, highlighting moderate heatwaves as the primary seasonal stressor. Peak heat occurred between 11:00 AM and 1:30 PM, coinciding with school dismissal, while shaded indoor areas maintained temperatures of 20–29 °C. Workshop areas were the hottest indoor spaces, with 66.5% of users experiencing thermal discomfort, whereas classrooms maintained comfort around 29.3 °C for 75% of students. Following the intervention, students’ climate-related knowledge improved significantly. Among proposed adaptation strategies, structural and technological measures dominated (> 70%), complemented by nature-based and behavioral interventions (~ 30%), while 75% of students employed active cooling measures and 35–40% used passive or green strategies. These results demonstrate the effectiveness of participatory, multi-scalar interventions in enhancing climate literacy, emotional engagement, and adaptive capacity among rural youth, while providing actionable insights for targeted heatwave adaptation in educational settings.

**Supplementary Information:**

The online version contains supplementary material available at 10.1007/s00484-026-03218-0.

## Introduction

Heatwaves are among the most critical manifestations of climate change, posing severe risks to human health, livelihoods, and ecosystems, particularly in tropical regions like Malaysia (Muhammad et al. 2024). Across Southeast Asia, heatwave frequency has increased by 1–3 events per decade, duration by 0.5–1.5 days per decade, and intensity by 0.2–0.5 °C per decade, with faster trends in minimum than maximum temperature, signifying more frequent warm nights and heightened health risks (Li et al. 2022; Yong and Chu 2023; Muhammad et al. 2024). While much attention focuses on urban heat stress (Hamed et al. 2024), rural communities are equally vulnerable due to limited adaptive capacity, inadequate infrastructure, and the absence of localized adaptation measures (Muhamad et al. 2025; How et al. 2025; Sa’adi et al. 2025c). Their reliance on outdoor and agricultural livelihoods further increases sensitivity to temperature extremes (Samsuddin et al. 2025). Thus, enhancing heatwave resilience demands a deeper understanding of local contexts and inclusive participation across all societal groups (Shamsuddin et al. 2025; Khan et al. 2026).

Youth in rural Malaysia constitute a highly vulnerable yet underrepresented group in climate adaptation planning (Saharrudin et al. 2018; Marty et al. 2025). Under the Malaysian Youth Societies and Youth Development Act 2007, youth are defined as individuals aged 15–30 years and represent approximately 30% of Malaysia’s population (around 10–12 million), with slightly more males than females (Hj Yeon et al. 2016). The census data indicate that those aged 15–24 accounted for about 20% of the population and had high literacy rates. Despite their demographic significance and educational attainment, empirical evidence suggests that rural youth possess limited adaptive capacity. For example, Saharrudin et al. (2018), in a study of 203 youth small-scale fishermen across four fisheries districts, namely, Batu Pahat (Johor), Pengkalan Chepa (Kelantan), Kuantan (Pahang), and Setiawan (Perak), reported adaptation capacity mean scores indicating relative weaknesses in employability (mean = 2.74), business scale (mean = 2.68), and livelihood diversification (mean = 2.71), where higher values represent greater adaptive capacity. These structural and economic constraints may restrict their ability to respond effectively to climate-related stressors. Nevertheless, rural youth remain marginally engaged as active stakeholders in adaptation planning processes, despite facing considerable physical and socio-economic risks. Heat stress increasingly disrupts their routines, learning environments, and health, undermining concentration and educational outcomes (Balakrishnan et al. 2025). Yet, youth possess creativity, adaptability, and social influence, positioning them as key agents for climate resilience (Tafon and Saunders 2025). Engaging them through participatory and co-creation approaches integrates their lived experiences, perceptions, and innovations into decision-making processes often dominated by adults and external experts (Cebrián et al. 2024).

Traditional climate adaptation in Malaysia has largely followed top-down approaches, focusing on scientific assessments and infrastructure solutions (Tam et al. 2023; Febriana et al. 2025; Ramli et al. 2025). While technically robust, these methods often neglect social and behavioral dimensions and fail to capture local complexities. Recent research highlights that effective adaptation requires place-based, inclusive, and participatory processes integrating scientific knowledge with community experience (Mat et al. 2023; Sa’adi et al. 2024d, 2025b; Ramli et al. 2025). Participatory mapping and co-adaptation frameworks have proven effective in bridging this gap, enabling shared learning, consensus building, and localized action by visualizing vulnerabilities, resources, and adaptive options at fine spatial scales (Lim et al. 2021).

Within participatory frameworks, the dotmocracy approach, a democratic decision-making method using colored stickers or dots, has gained traction as a simple yet effective tool to prioritize community-driven solutions (Mugari et al. 2025; Siganul and Puspitasari 2025). It enables collective expression of preferences, enhancing transparency, inclusivity, and critical negotiation skills. When combined with data-driven facilitation and visual storytelling (Perdana et al. 2025), dotmocracy offers an accessible platform for youth engagement in climate resilience planning (Donatuto et al. 2019; Kimbell et al. 2025), transforming adaptation discussions from abstract concepts into tangible, community-centered actions.

This study presents a youth-led, spatio-temporal heatwave co-adaptation mapping initiative in Segamat, Johor, Malaysia, engaging rural high school students to identify and prioritize adaptation measures through a structured participatory process. The approach combines emotion-based reflection, gamification, climate literacy activities, and spatiotemporal dotmocracy mapping to capture students’ perceptions of heatwave risks and preferred responses. Specifically, this study addresses the following research questions: (1) How do rural youth perceive and experience heatwave risks within their school environment? (2) What adaptation measures do they identify and prioritize through a participatory dotmocracy-based mapping process? (3) How can youth-led spatio-temporal co-adaptation mapping enhance climate literacy, adaptive capacity, and inclusive local adaptation planning?

By visualizing their school environment, discussing feasible solutions, and voting on priorities, students collaboratively produced a localized adaptation map addressing immediate and long-term needs. The study advances participatory climate adaptation by positioning youth as knowledge co-producers, demonstrating how integrating local knowledge, democratic decision-making, and spatial visualization can enhance climate literacy, adaptive capacity, and collective ownership. Findings provide guidance for incorporating youth perspectives into local adaptation planning and for replicating participatory mapping in other climate-vulnerable communities in Malaysia and beyond.

## Study setting and participants

**Fig. 1:**
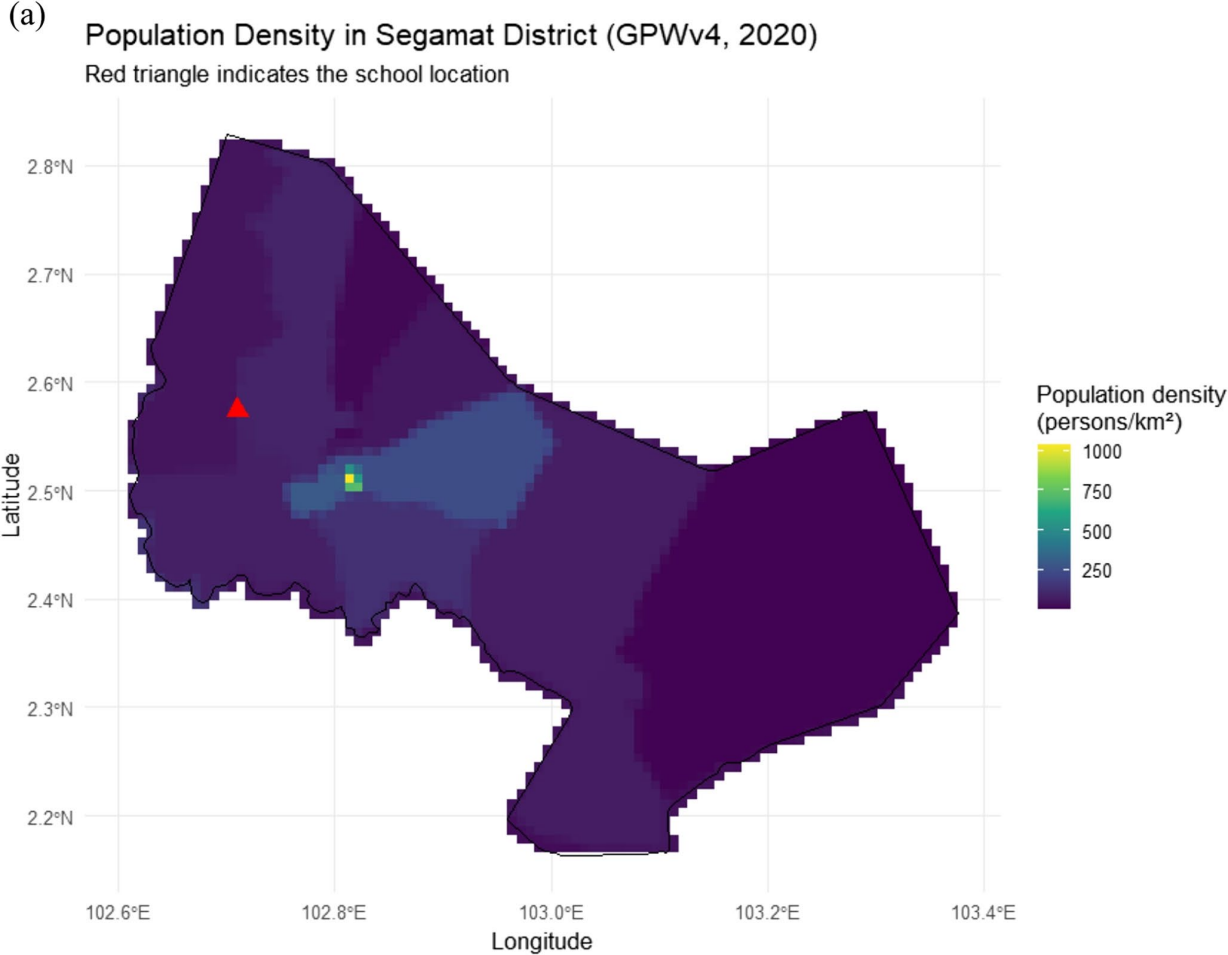

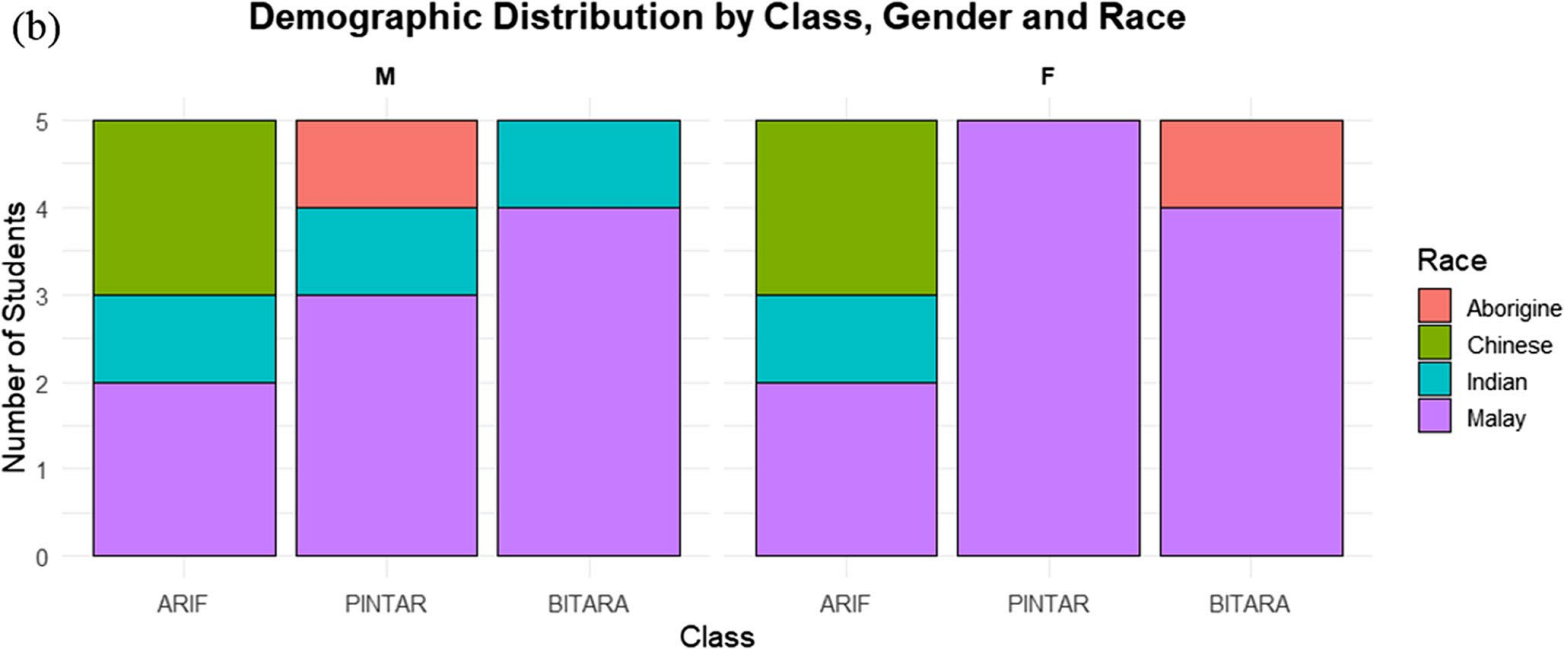
(**a**) Spatial distribution of population density in Segamat District, Johor, Malaysia, derived from the GPWv4 (2020) dataset at ~ 1 km resolution and clipped to the district boundary. (**b**) Demographic distribution of students across classes (Arif, Pintar, Bitara) by gender (M = male; F = female) and race. Bars represent the total number of students in each class, with colors indicating racial groups (Chinese, Malay, Indian, Aborigine)

The study was conducted at a government secondary school in rural Segamat, Johor, Malaysia, a district with a tropical climate and rising exposure to extreme heat (Houmsi et al. 2023a). Based on ground observation, the school is situated within a small-town setting and is predominantly surrounded by oil palm plantations and forested areas, indicating a largely rural land-use context with limited dense urban development. Spatial analysis of the GPWv4 population density (CIESIN 2018) further shows that Segamat covers approximately 6,206.89 km², with population density ranging from 1.89 to 1,039.76 persons km⁻² (mean = 63.88 persons km⁻²; SD = 65.67), highlighting substantial spatial heterogeneity in settlement patterns across the predominantly rural district (Fig. 1a). Thirty Form Three students (~ 15 years old; equivalent to the final year of lower secondary school in Malaysia, marking the end of Form 1–3 before progressing to upper secondary school) from diverse ethnic, religious, and socio-economic backgrounds participated. Participants were purposively selected by teachers to ensure inclusive representation and organized into six heterogeneous groups of five to support collaborative engagement. As shown in Fig. 1(b), demographically, students from 3 different class, namely, Arif, Pintar, and Bitara participate in the program. The overall student demographics indicate that Malay students constitute the majority (*n* = 18, 60%), followed by Chinese (*n* = 4, 13%), Indian (*n* = 4, 13%), and Aborigine students (*n* = 2, 7%). Gender distribution was relatively balanced, with 15 males and 15 females. Among female students, Malay individuals were most prominent (*n* = 11, 73% of females), while male students were more evenly distributed across Malay (*n* = 9, 60%), Chinese (*n* = 2, 13%), Indian (*n* = 2, 13%), and Aborigine (*n* = 2, 13%) groups.

According to the Department of Statistics Malaysia, the citizen population estimate for Segamat in 2023 was 186,200, comprising Malays (103,600), Chinese (63,000), Indians (15,700), and others (3,700). This broader demographic structure indicates a Malay-majority district with substantial Chinese and smaller Indian and other communities. Although the sample includes multiple racial groups, Malay students dominate the participant composition (*n* = 18, 60%), followed by Chinese (*n* = 4, 13%), Indian (*n* = 4, 13%), and Aborigine students (*n* = 2, 7%), which generally mirrors the district-level ethnic distribution reported by the national statistics, albeit with slight underrepresentation of Chinese students and limited participation from minority groups. Gender distribution was balanced, with 15 males and 15 females. This composition highlights the predominance of Malay students and the minimal representation of minority groups such as Aborigine students, which is important for interpreting participation and engagement in the youth-led heatwave co-adaptation mapping initiative. At the school’s request and application, and with formal approval from the MOSTI STI 100³ program supervisory body (Academy of Sciences Malaysia), the structured workshop enabled students to explore and prioritize context-specific heatwave adaptation strategies through guided learning, group mapping, and participatory decision-making tools. Parental consent and school approval were obtained in accordance with Ministry of Education guidelines and the ethical oversight framework of the MOSTI STI 100³ program, ensuring adherence to standards for research and activities involving minors.

## Methodology

Table 1 outlines the structured sequence of activities implemented in the youth-led heatwave co-adaptation program, detailing each step from pre-survey to post-survey. The program began at 09:45 AM with a pre-survey to capture baseline knowledge and perceptions of climate change and heatwave risks. From 09:50 to 10:15 AM, students participated in a mini talk on climate change and heatwave, which included dissemination of an interactive tri-brochure, an emoji-based reflection on emotions toward climate change, and a quiz to reinforce learning. The main participatory activity, heatwave co-adaptation mapping, took place from 10:15 to 11:30 AM, with each student group supported by one facilitator to guide the exercises and discussions. During this session, students listed personal strategies to stay cool at school, identified periods of thermal comfort and peak heat, and collaboratively mapped three heat-prone and three cooler areas within the school. For each identified hotspot, groups proposed strategies to mitigate heat exposure or enhance comfort. Throughout this activity, students were encouraged to contribute to the “Our Promise Tree” pledge. From 11:30 to 12:00 PM, students presented their group findings, followed by a post-survey from 12:00 to 12:05 PM to capture changes in knowledge and perceptions.

**Table 1:** Timeline of the youth-led heatwave co-adaptation mapping program, detailing the sequence of activities, sub-steps, and engagement strategies used from pre-survey to post-survey

Time	Activity	
09:45 − 09:50AM	Pre-survey	
09:50 − 10:15AM	Mini Talk on climate change and heatwave	1. Dissemination of interactive tri-brochure. 2. Emoji & Emotions: Your Reactions to Climate Change. 3. Quiz.
10:15 − 11:30AM	Heatwave co-adaptation mapping	1. Students listed unique strategies they use to stay cool at school. 2. Students identified periods of relative thermal comfort and peak heat. 3. Groups identified and marked three heat-prone areas within the school. 4. Groups identified and marked three cooler areas within the school. 5. For each hotspot, groups proposed strategies to reduce heat exposure or enhance comfort. “Our Promise Tree” pledge was disseminated throughout this activity.
11:30 − 12:00PM	Group presentation	
12:00–12:05PM	Post-survey	

### Local characterization of Tmax and heatwave characteristic

At the initial stage of the study, the impacts of climate change in the study area were investigated, which identified heatwave occurrence as one of the key climate-related stressors affecting the local environment in Segamat as reported by Suparta and Mohd Yatim (2019); Muhammad et al. (2024); Sa’adi et al. (2024c). To contextualize students’ perceptions of heat exposure, daily maximum temperature (Tmax) records (1950–2022) were extracted from the ERA5 reanalysis dataset for the grid point nearest the school (2.574°N, 102.710°E) (Chua and Safari 2025) via the Copernicus Climate Data Store. Distances to all grid points were computed using the Haversine formula (Alkodri et al. 2025), and the relevant Tmax time series was used to visualize heatwave conditions during Mini Talk and to explicitly support the data-driven participatory mapping framework adopted in this study. A linear regression on daily Tmax estimated long-term warming, while monthly statistics (mean, variance, skewness, kurtosis) characterized seasonal variability under the region’s monsoonal climate (Sa’adi et al. 2024a; Kemarau et al. 2026).

A single-variable, locally recognised heatwave definition based on daily Tmax thresholds established by the Malaysian Meteorological Department (Wahab and Razak 2024) was used, as it is easily understood by youth participants. Daily Tmax was classified into four levels, namely, Level 0 (< 35 °C, non-heatwave), Level 1 (35–37 °C, caution), Level 2 (37–40 °C, heatwave), and Level 3 (> 40 °C, extreme heatwave), each sustained over ≥ 3 consecutive days. This straightforward, locally relevant definition ensured that participatory mapping and student engagement were grounded in heat risk conditions meaningful to the community, rather than relying on more complex indices incorporating multiple variables such as Heat Index (Kamal et al. 2022) or Wet Bulb Globe Temperature (Houmsi et al. 2023b). This localized diagnostic was intentionally incorporated to ground student discussions in observed heat risk conditions rather than generalized climate information. This framework enabled spatiotemporal analysis of heatwave severity, facilitating comparison between objective temperature extremes and students’ subjective experiences, thereby directly informing the prioritization of youth-identified co-adaptation measures during the participatory workshops.

### Program survey

To evaluate the intervention’s impact on students’ climate knowledge and awareness, a pre- and post-assessment design was implemented. Such designs provide baseline and outcome measures, enable statistical testing of individual changes, identify effective program components and subgroups, and support evidence-based program improvement (Portela Dos Santos et al. 2022). The survey questions were specifically developed for this study and were not modified from previously published instruments, ensuring they were contextually relevant, age-appropriate, and aligned with the learning objectives of the school-based heatwave co-adaptation program. The pre- and post-assessment as provided in supplementary materials 1 and 2 comprised three sections: (i) knowledge questions (B1–B5), (ii) awareness, interest, and self-efficacy items (C1-C7), and (iii) open-ended reflections (D1–D3). Knowledge was assessed via five multiple-choice questions on weather–climate distinctions, non-climatic impacts, carbon footprint, climate action, and water-related impacts.

Responses were coded dichotomously, with pre- and post-intervention changes analyzed using McNemar’s test (Pembury Smith and Ruxton 2020; Meixner et al. 2024). McNemar’s test was selected to assess pre- and post-intervention changes in dichotomous knowledge responses because it is specifically designed to test paired nominal data, evaluating whether the proportion of correct responses changed significantly after the intervention. To control for multiple comparisons across knowledge and awareness items, Bonferroni correction was applied to the McNemar’s p-values and the significance thresholds of paired t-tests and Wilcoxon signed-rank tests. This approach is widely used in educational and intervention studies to measure individual-level changes in binary outcomes. For example, Sanz-Mas et al. (2024) reported that students correctly identifying climate-weather distinctions increased from 54% pre-intervention to 82% post-intervention (*p* < 0.001), while Torres et al. (2023) found that Group I participants significantly improved in two of four communication skills, with correct responses rising from 46% to 78% (*p* < 0.05), whereas Group II showed no significant change.

Awareness, interest, and self-efficacy items employed a five-point Likert scale, with paired t-tests and Wilcoxon signed-rank tests applied to detect mean differences and account for non-normal distributions. Bonferroni-adjusted significance thresholds were used to reduce the risk of Type I errors when evaluating multiple awareness items. Analyses were conducted in R (*p* < 0.05). Qualitative data from open-ended responses captured students’ understanding of climate change, personal mitigation actions, and school-level strategies. Thematic content analysis involved open coding and grouping into broader themes reflecting scientific understanding, behavioral intent, and contextual relevance. Combining quantitative and qualitative analyses enabled triangulation, providing a comprehensive assessment of short-term cognitive, perceptual, and motivational changes, and informing curriculum refinement for climate education (Fukaya et al. 2024; McShane et al. 2024).

### Mini talk and heatwave tri-brochure

The participatory program began with a structured Mini Talk to build students’ foundational understanding of climate change, heatwaves, and adaptation strategies. Structured workshops have been shown to enhance youth comprehension of climate issues, link climate to social outcomes, and foster community-level action Baldwin et al. (2022; Larose et al. 2022). The session had two components. The first introduced core climate concepts, emphasizing anthropogenic drivers, global and national impacts, and heatwaves as a critical hazard. ERA5 Tmax and local heatwave data were visualized to contextualize risks for the school environment, illustrating implications for health, education, and infrastructure. The second component focused on adaptation and personal preparedness, distinguishing adaptation from mitigation and highlighting practical strategies to reduce heat stress in school settings. This foundational session prepared students to actively participate in the subsequent dotmocracy co-adaptation mapping, enabling informed identification of vulnerabilities and locally relevant preparedness measures.

An interactive tri-fold brochure, “Reflection: My Climate Voice”, was developed and distributed to each student as a core component of the Mini Talk intervention (Supplementary Material 3). Such structured and interactive materials have been shown to enhance youth climate knowledge and foster pro-environmental action Janney et al. (2024; Gupta et al. 2025). Designed with youth-friendly visuals, concise text, and gamified elements, the brochure guided students through key concepts, including local Tmax visualizations, identification of extreme climatic locations in Malaysia, climate processes via photovoice, school co-adaptation mapping, heatwave definitions, monitoring equipment, and historical heatwave frequency. Clear, accessible language, colorful icons, and relatable imagery supported comprehension, reflection, and engagement. Facilitators used the brochure during mini-lectures, quizzes, and mapping exercises, linking knowledge acquisition to action-oriented reflection. By integrating interactive, visual, and localized content with hands-on activities, the brochure reinforced climate literacy, promoted critical thinking, and encouraged application of adaptation practices beyond the workshop Ramos et al. (2024; Koç and Kanadlı 2025).

### Participatory visual engagement through emoji reflection and gamification

The workshop began with the participatory activity “Emoji & Emotion,” prompting students to reflect on and express their emotional responses to climate change. Linking climate information with emotions has been shown to enhance cognitive complexity, connect impacts to societal consequences, and motivate personal and collective action Oberauer et al. (2022; Levesque and Rocque 2024). Six photographs depicting stages of climate change, from greenhouse gas emissions to impacts such as forest fires and floods, and climate action, were used as visual prompts (Supplementary Material 4). Each student drew an emoji on a sticker to represent their emotional response, and photos rotated among groups so that each contained ten unique reactions. A follow-up gallery walk allowed discussion and reflection, leveraging evidence that visual and affective pedagogies enhance engagement, memory, and behavioral intentions (Kaufmann et al. 2023). This activity created a safe space for emotional expression, fostered empathy, and prepared students for deeper discussions on local vulnerabilities and adaptation strategies.

Following the Emoji & Emotion activity, students engaged in a climate process gamification exercise to strengthen conceptual understanding through collaborative problem-solving. Using the same six photographs depicting the climate change process (Supplementary Material 4), each group arranged the shuffled images in chronological order, discussing the cause-effect flow from greenhouse gas emissions to climate impacts and solutions. Groups built consensus, followed by a plenary session where facilitators clarified misconceptions and explained the scientifically accepted sequence. This gamified approach fostered systems thinking, peer dialogue, and narrative construction, making abstract climate science tangible for students with limited prior exposure. Gamification has been shown to enhance motivation and learning outcomes in sustainability education (Yang et al. 2024), serving as a cognitive bridge to the subsequent applied adaptation mapping exercises.

### Dotmocracy co-adaptation mapping and solutions

The third workshop session adapted the co‑adaptation mapping approach (Sa’adi et al. 2025a), to a school setting, focusing on heatwave-related vulnerabilities. Six student groups engaged in structured participatory exercises to assess temporal and spatial heat exposure and identify context-specific adaptation solutions. Each group used a school floor map, pens, and red/green stickers to complete five guiding tasks:

1. Personal adaptation (Q1): Students listed unique strategies they use to stay cool at school, highlighting diverse coping practices.
2. Coldest and hottest times (Q2): Students identified periods of relative thermal comfort and peak heat, capturing daily temperature perceptions.
3. Hotspot identification (Q3): Groups marked three heat-prone areas with red stickers, based on sunlight, ventilation, and discomfort.
4. Cool spot identification (Q4): Groups marked three cooler areas with green stickers as potential refuge zones.
5. Co-adaptation recommendations (Q5): For each hotspot, groups proposed strategies to reduce heat exposure or enhance comfort.

Sessions concluded with group presentations, enabling students to articulate reasoning, share perspectives, and receive facilitator feedback. Outputs were collected and analyzed to identify patterns in perceived hotspots, cool zones, and adaptation proposals. The exercise captured student perceptions, coping strategies, and visually highlighted areas for targeted adaptation, fostering agency and participatory climate resilience planning.

## Results and discussion

### Local characteristic of maximum temperature and heatwave assessment

**Fig. 2:**
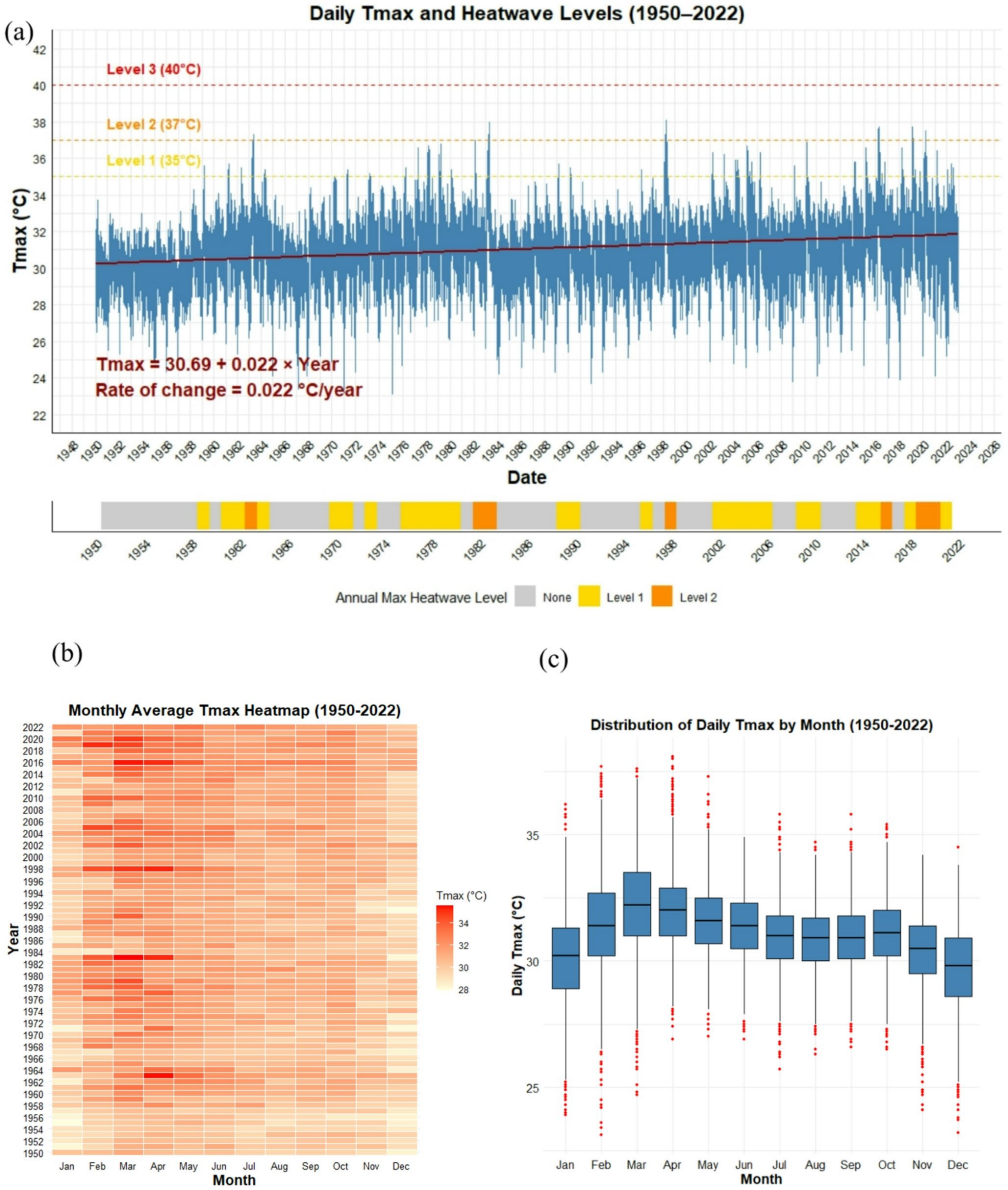
(**a**) Daily Tmax at from 1950–2022 with heatwave thresholds overlaid (Level 1: 35 °C, Level 2: 37 °C, Level 3: 40 °C). The solid red line represents the linear regression trend, with the corresponding equation and rate of change (°C/year) shown at the bottom-left. The lower panel (**b**) displays annual maximum heatwave levels using a tile-based stripe plot, highlighting the temporal progression and intensity of heatwave exposure across the study period, and (**c**) The boxplot of monthly temporal pattern of Tmax. The median is shown as a horizontal line within each box, and red dots denote outliers

Linear regression of daily Tmax representing the school’s vicinity (1950–2022) revealed a significant warming trend (Tmax = − 13.14 + 0.0222 × Year; t = 46.86, *p* < 2 × 10⁻¹⁶), corresponding to an average increase of 0.022 °C/year (~ 0.22 °C/decade) (Fig. 2(a-b). Despite the relatively low explanatory power (R² = 0.076), which reflects the high day-to-day variability typical of tropical climates (residuals − 7.86 to 7.02 °C), the trend provides a robust local warming signal. Consistent with the study’s participatory focus, this localized evidence was used to contextualize heat risk for students during the Mini Talk and subsequent mapping exercises, aligning with regional observations of rising Tmax and heatwave frequency in Peninsular Malaysia (Muhammad et al. 2024; Sa’adi et al. 2024c).

Heatwaves (Tmax > 35 °C) were rare: of 26,663 days, 377 (1.41%) were Level 1 (35–36.9 °C), 27 (0.10%) Level 2 (37–40 °C), and none exceeded 40 °C (Level 3). Level 1 days first appeared in the late 1950 s, with episodic clustering in years such as 1983, 1998, 2005, and 2016, while Level 2 events remained extremely rare. Peaks corresponded with El Niño years, consistent with ENSO-driven modulation of drought and heatwave frequency and intensity (Sa’adi et al. 2022, 2024b; Wu et al. 2024; Saadi et al. 2025). Although most years were free from severe heat stress, recent decades show modest increases in both frequency and intensity of Level 1–2 events; early decades (1950–1970 s) typically had < 5 Level 1 days/year, whereas 1998, 2016, and 2019–2020 recorded 20–44 Level 1 days and occasional Level 2 events. These findings underscore gradual warming, episodic heatwave clustering, and the need for adaptive strategies in schools to prepare for more frequent heat stress in the future (Zeder and Fischer 2023). Building on this evidence, the derived diagnostics were used to identify priority heat periods for discussion with participants, thereby supporting evidence-based co-adaptation planning in the school setting.

Analysis of monthly Tmax (1950–2022) reveals clear seasonal patterns linked to the regional monsoon cycle (Fig. 2c; Table 2). During the Northeast Monsoon (NEM, November-March), mean Tmax ranged 29.7–32.2 °C, peaking in March (37.6 °C). Variance was highest in January–February (3.45–3.8 °C), indicating greater day-to-day fluctuations, while the Southwest Monsoon (SWM, May-September) showed lower variance (1.61–1.66 °C), reflecting more stable temperatures (Sa’adi et al. 2026). Inter-monsoon months (April, October) exhibited intermediate variability. Positive skewness in April (0.458) indicates higher likelihood of extreme highs, whereas negative skewness in November–December (−0.533 to −0.399) suggests cooler days; kurtosis was generally low, peaking at 0.8 in April, highlighting moderate probability of extremes. These results indicate that temperature extremes cluster at the end of NEM and inter-monsoon months, while SWM remains comparatively stable, consistent with patterns observed in humid tropical regions (Houmsi et al. 2023a; Jackson et al. 2025). Extreme heat days (> 35 °C) also show strong seasonality. March had the highest frequency (160 days, 7.07%), followed by April (98 days, 4.47%) and February (71 days, 3.44%), while mid- and late-year months recorded few or no extreme days (e.g., September 2 days, 0.09%; October 3 days, 0.13%). January recorded 10 days (0.44%), and May–July 4–18 days (0.18–0.8%). This seasonal evidence was incorporated into the participatory sessions to help students recognize periods of elevated heat risk within the school calendar. In particular, the analysis highlights that late NEM to inter-monsoon months (February-April) represent the peak period for heat exposure, providing a critical reference for planning public health measures and school-based adaptation. These observed patterns are consistent with regional studies reporting increasing Tmax and higher frequency of hot days across Peninsular Malaysia (Tan et al. 2021).

**Table 2:** Monthly statistical summary (minimum, maximum, average, variance, skewness, and kurtosis) of daily Tmax from 1950 to 2022 at SMK Dato’ Ahmad Arshad

Month	Min	Max	Avg	Variance	Skewness	Kurtosis
Jan	23.9	36.2	30.1	3.45	−0.167	0.372
Feb	23.1	37.7	31.5	3.80	−0.128	0.523
Mar	24.7	37.6	32.2	3.66	−0.152	0.311
Apr	26.9	38.1	32.1	2.53	0.458	0.800
May	27	37.3	31.6	1.86	0.119	0.241
Jun	26.9	34.9	31.4	1.64	−0.156	−0.079
Jul	25.7	35.8	30.9	1.66	−0.233	0.292
Aug	26.3	34.7	30.8	1.63	−0.111	−0.154
Sep	26.6	35.8	30.9	1.61	−0.080	0.092
Oct	26.5	35.4	31.0	1.83	−0.174	0.110
Nov	24.1	34.2	30.4	2.01	−0.533	0.661
Dec	23.2	34.5	29.7	2.69	−0.399	0.065

**Fig. 3:**
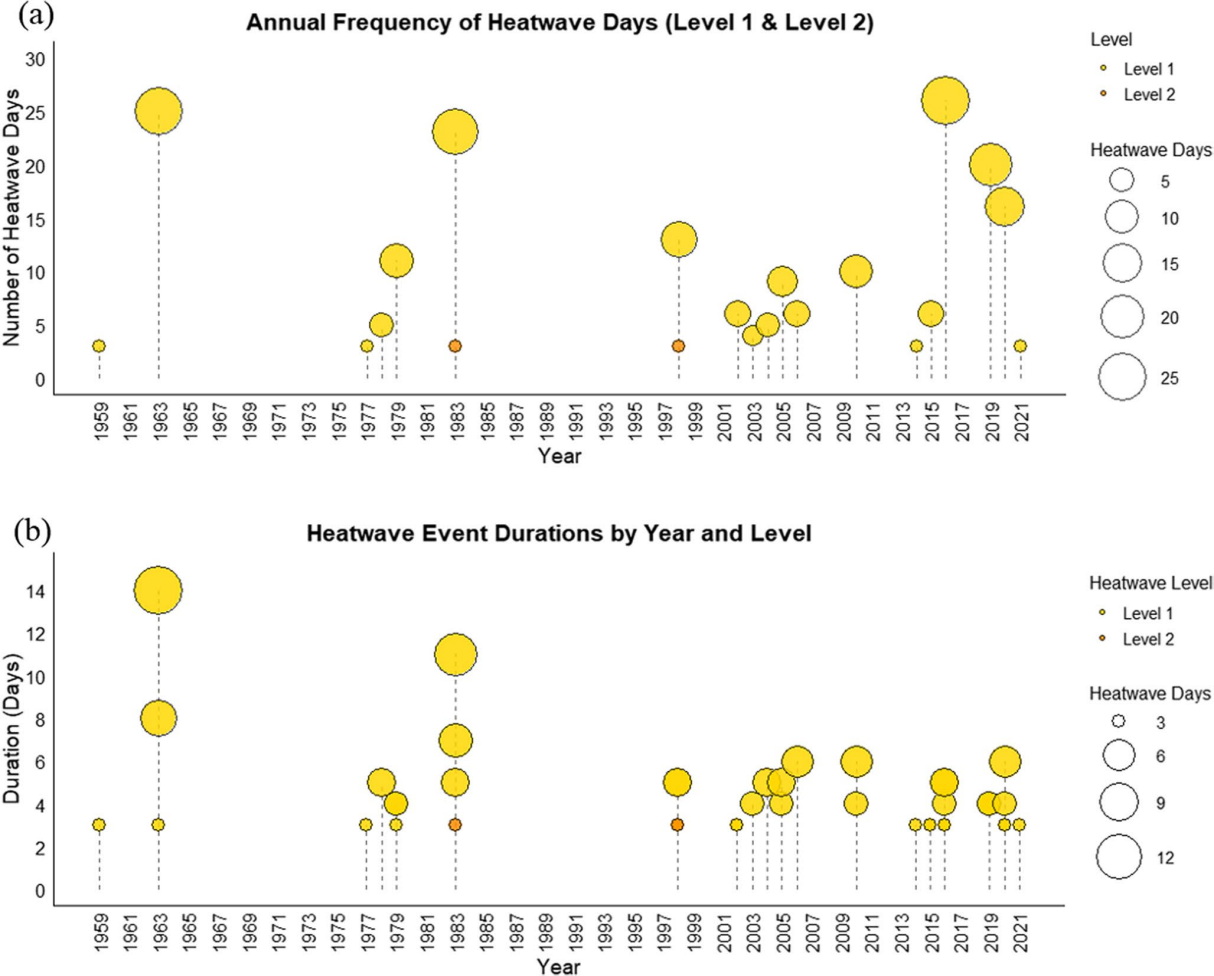

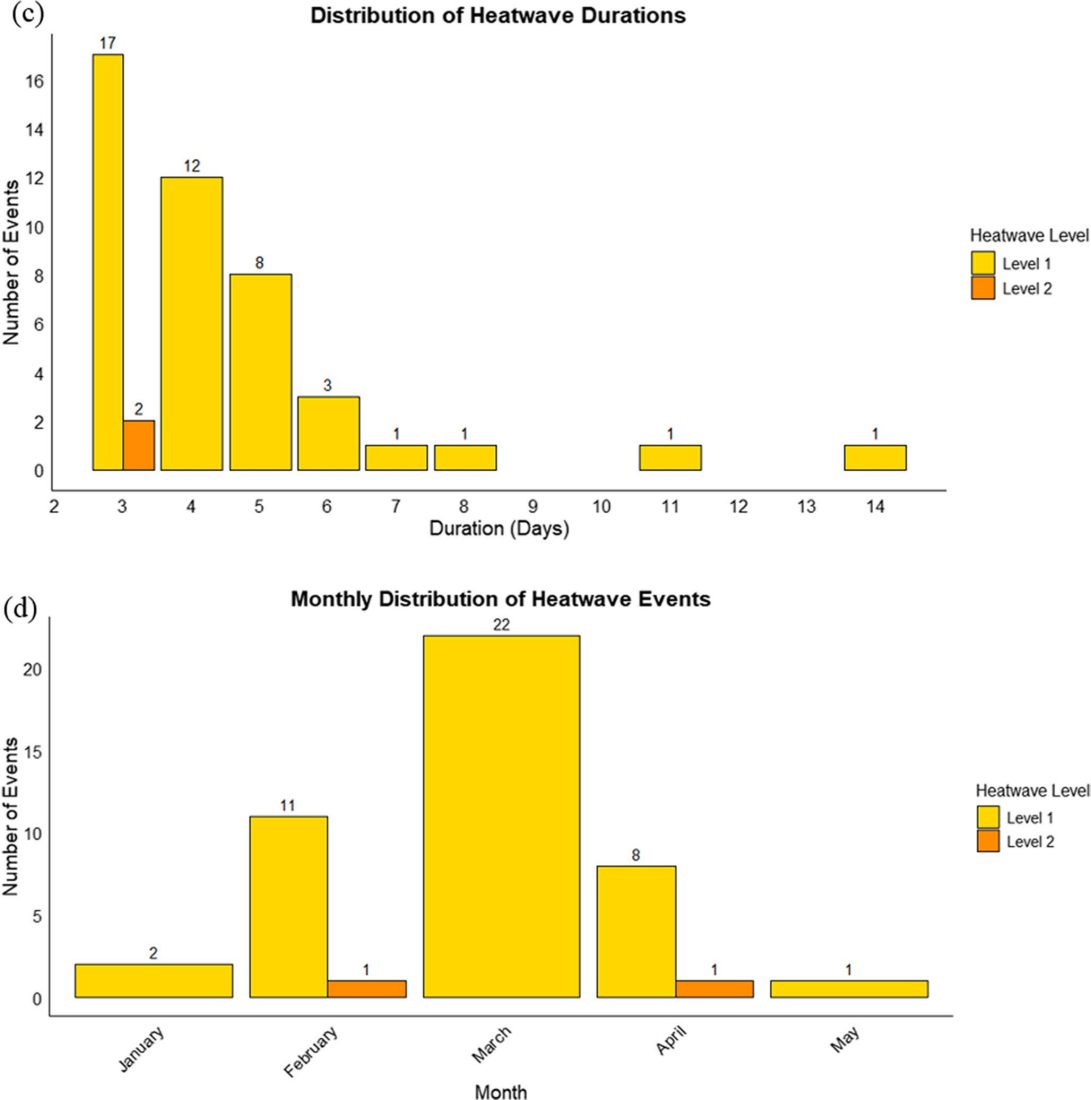
Bubble lollipop plot of the (**a**) annual frequency and (**b**) event’s duration in days of heatwave, respectively,, by severity level (Level 1 in yellow and Level 2 in orange) recorded at SMK Dato’ Ahmad Arshad, Segamat, Johor. Grouped bar plot showing (**c**) the distribution of heatwave event durations and (**d**) the number of heatwave events per month, respectively, by severity level (Level 1 in yellow and Level 2 in orange)

Analysis of annual heatwaves shows that Level 1 events (> 35 °C) were far more frequent than Level 2 (> 37 °C) in the study area (Fig. 3a). Over 73 years, 197 Level 1 days occurred versus 6 Level 2 days, with Level 1 peaking at 26 days in 2016 and Level 2 at 3 days in 1983. Level 1 events were also longer and more frequent (44 events, mean 4.48 days/event, maximum 14 days in 1963) compared to Level 2 (2 events, mean 3 days/event, maximum 3 days in 1983) (Fig. 3b). Including this detailed Tmax and heatwave analysis provides a localized, evidence-based context that directly informed the participatory activities in this study, allowing students to engage with observed heat risk. Moderate, prolonged heatwaves dominate seasonal heat stress, posing significant risks to vulnerable populations and health systems (Kamal et al. 2022; Sapari et al. 2023; Kemarau et al. 2025), highlighting the need to account for both frequency and duration in adaptation planning.

Event duration and monthly occurrence (Fig. 3c-d) further illustrate distinct characteristics. Most Level 1 events were short: 17 events lasted 3 days, 12 lasted 4 days, and 8 lasted 5 days; longer events were rare. Level 2 events were extremely scarce, with only two 3-day events. Level 1 events clustered in the late NEM and inter-monsoon periods, peaking in March (22 events), followed by February (11) and April (8), while the SWM period (May–September) saw minimal activity. Level 2 events occurred only in February and April. These temporal patterns were incorporated into the participatory sessions to help students recognize periods of elevated heat risk and guide their co-adaptation mapping, emphasizing early-year monitoring and preparedness. These patterns align with regional surface temperature trends (Ahmad Kamal et al. 2019) and emphasize the importance of early-year monitoring and preparedness for heat stress in the region.

Analysis of intense heatwave events shows that Level 1 events dominated in both frequency and duration, with 46 events recorded versus only 2 Level 2 events (Supplementary Material 5). Level 1 durations ranged from 3 to 14 days, most commonly 3 days (17 events), followed by 4 days (12 events) and 5 days (8 events); longer events were rare. Level 2 events were shorter, both lasting 3 days, with maximum intensities of 38 °C (April 1983) and 37.3 °C (February 1998). Temporal distribution exhibited clear seasonality, concentrated in late NEM and inter-monsoon months (January–April), peaking in March–April, consistent with historical Tmax peaks. Notable Level 1 events include March 1963 (8 days) and April 1963 (14 days), March 1983 (7 days, 36.7 °C), and March 2020 (6 days, 36.3 °C). These findings confirm that early-year months are most prone to heatwaves, typically 3–5 days in duration with occasional extremes exceeding 10 days, highlighting the relevance of the heatwave diagnostics for seasonal preparedness and evidence-based co-adaptation planning in the school setting.

### Student cognitive, perceptual, and emotional responses to the heatwave adaptation program

**Fig. 4:**
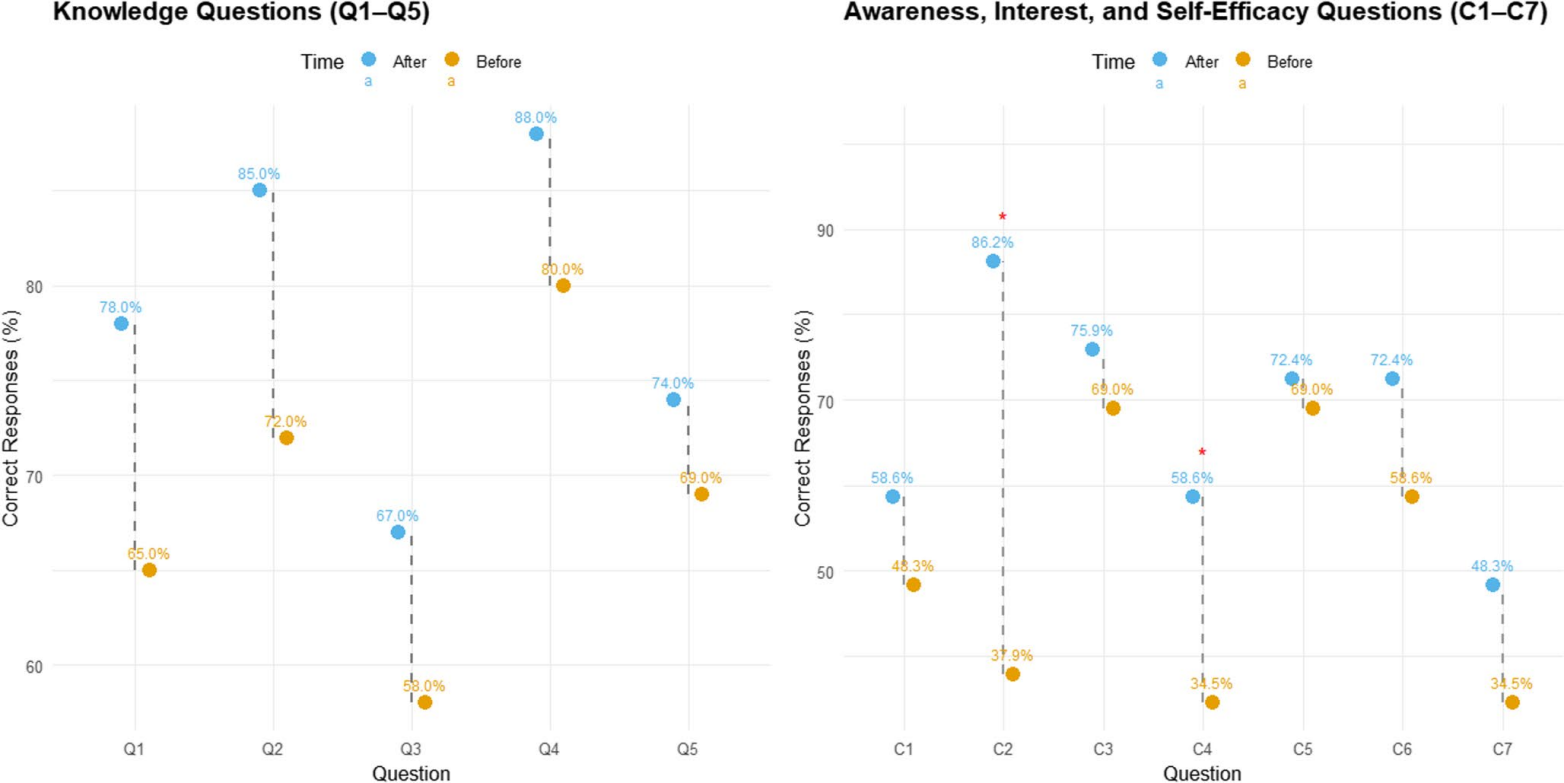
Pre- and post-intervention comparison of student knowledge and awareness responses. (Left) Knowledge questions (B1-B5) show the percentage of correct responses before and after the intervention. (Right) Awareness questions (C1-C7) display the percentage of correct/agree responses, with red asterisks indicating statistically significant changes after Bonferroni-adjusted McNemar tests (*p* < 0.05). Dashed lines connect paired pre- and post-intervention responses for each question

Figure 4 presents a comparative analysis of students’ performance in both knowledge, awareness, interest, and self-efficacy assessments, illustrating measurable changes before and after the intervention. The response can be obtained through the Mendeley data repository (Sa’adi 2025). McNemar’s test with Bonferroni correction was applied to assess changes in knowledge before and after the intervention across five climate-related questions (B1-B5) (Supplementary Material 6). The proportion of correct responses increased for all questions following the intervention: Q1 improved from 72.41% to 96.55%, Q2 from 65.52% to 93.10%, Q3 from 89.66% to 93.10%, Q4 from 68.97% to 93.10%, and Q5 from 51.72% to 65.52%. The unadjusted McNemar’s p-values indicated nominal statistical significance for Q1 (*p* = 0.046), Q2 (*p* = 0.027), and Q4 (*p* = 0.023), whereas Q3 (*p* = 1.000) and Q5 (*p* = 0.386) showed no significant changes. After applying the Bonferroni adjustment to account for multiple comparisons, none of the differences reached the adjusted significance threshold (Q1: 0.228; Q2: 0.135; Q3: 1.000; Q4: 0.117; Q5: 1.000). These results show an observable increase in correct responses across all knowledge items. However, after adjusting for multiple comparisons using the Bonferroni method, the improvements did not reach statistical significance. The lack of significance may be attributed to the relatively small sample size (*n* = 30), which limits statistical power, and the already high baseline scores for some questions, leaving limited room for measurable improvement. Despite this, the upward trend in correct responses suggests a positive effect of the intervention, consistent with previous studies reporting enhanced climate knowledge, awareness, interest, and self-efficacy following educational programs (*P* < 0.000) (K. Ghazy and M. Fathy 2023).

Changes in students’ awareness, interest, and self-efficacy were evaluated across seven items (C1-C7) using a paired design, with pre- and post-intervention responses analyzed via paired t-tests, Wilcoxon signed-rank tests, and McNemar’s test for dichotomized agreement (Likert 4–5 = 1, else 0) (Table 3). Significant increases were observed for C2 (“I understand that climate change can affect the quality and quantity of water”) and C4 (“I am aware of the importance of heatwave risk mapping in addressing climate change”), with paired t-test, Wilcoxon, and Bonferroni-corrected p-values all below 0.05. Additionally, C7 (“I believe I can become an agent of change in my school or community”) showed a significant change under the Wilcoxon test, highlighting enhanced self-efficacy following the intervention. Items C1, C3, C5, and C6 demonstrated positive trends, with mean scores and agreement percentages increasing post-intervention. However, these changes did not reach statistical significance after adjustment for multiple comparisons. Specifically, C1 increased from 48.3% to 58.6%, C3 from 69.0% to 75.9%, C5 from 69.0% to 72.4%, and C6 from 58.6% to 72.4%, suggesting modest gains in awareness, interest, and STEM-related motivation.

**Table 3:** Comparison of mean awareness scores before and after the intervention (C1-C7) with corresponding paired t-test and Wilcoxon signed-rank test *p*-values

Question	Mean				Correction		
	Before	After	t-test *p*	Wilcox *p*	Before	After	*p*-value
C1. I am aware that my actions can impact the environment and climate change.	3.52	3.72	0.495	0.351	48.3	58.6	0.51
C2. I understand that climate change can affect the quality and quantity of water.	3.1	4.28	0.000*	0.001*	37.9	86.2	0.00*
C3. I will save electricity to reduce the risks of climate change.	3.79	4.17	0.054	0.053	69.0	75.9	0.68
C4. I am aware of the importance of heatwave risk mapping in addressing climate change.	3.14	3.83	0.011*	0.015*	34.5	58.6	0.02*
C5. I am confident that applying knowledge in STEM can help reduce the risks of climate change.	3.97	4.03	0.778	0.749	69.0	72.4	1.00
C6. I am interested in exploring STEM fields to solve environmental problems.	3.79	4.21	0.103	0.146	58.6	72.4	0.22
C7. I believe I can become an agent of change in my school or community.	2.93	3.45	0.070	0.029*	34.5	48.3	0.29

STEM means Science, Technology, Engineering & Math. Bold asterisk means significant changes

Open-ended responses (D1-D3) supported these quantitative trends, revealing more accurate and detailed definitions of climate change, clearer articulation of personal mitigation actions (e.g., energy saving, recycling, water conservation), and structured school-level initiatives (recycling programs, tree planting, agro-parks, solar panels). These qualitative findings reflect enhanced scientific understanding, motivational engagement, and solution-oriented thinking, consistent with prior studies linking knowledge gains to pro-environmental behavior Akakpo et al. (2024). Overall, the intervention produced statistically significant improvements in students’ awareness of climate–water interactions and heatwave risk mapping, as well as self-efficacy to act as change agents, while also fostering positive trends across other awareness and interest measures, indicating the program’s effectiveness in strengthening both cognitive and motivational dimensions of climate education.

Participants’ emotional responses during a gamified climate change exercise were captured via emoji-based reflections (Table 4), showing stage-specific affective reactions. At the early stage of greenhouse gas emissions, participants mainly expressed negative or mixed emotions (sadness, nervousness, anger, sickness). In the heat-trapped stage, anxiety, crying, surprise, and sickness increased. The hot stage elicited anxious, nervous, neutral, and occasional positive reactions (happiness, smiling). At the fire stage, distress dominated (dizziness, disappointment, sadness, worry, shock, crying), while the flood stage showed similar negative emotions with occasional smiles. The action stage shifted to primarily positive emotions (happiness, smiling, surprise, love), indicating hope and motivation. Open-ended responses confirmed this trend: emotions evolved from negative/neutral in early and high-impact stages to positive/hopeful in the action stage, consistent with Garfin et al. (2024), who report that regulated negative climate emotions predict pro-environmental behaviour. These results demonstrate that gamification effectively captures evolving emotional perceptions and fosters engagement with climate action.

**Table 4:**
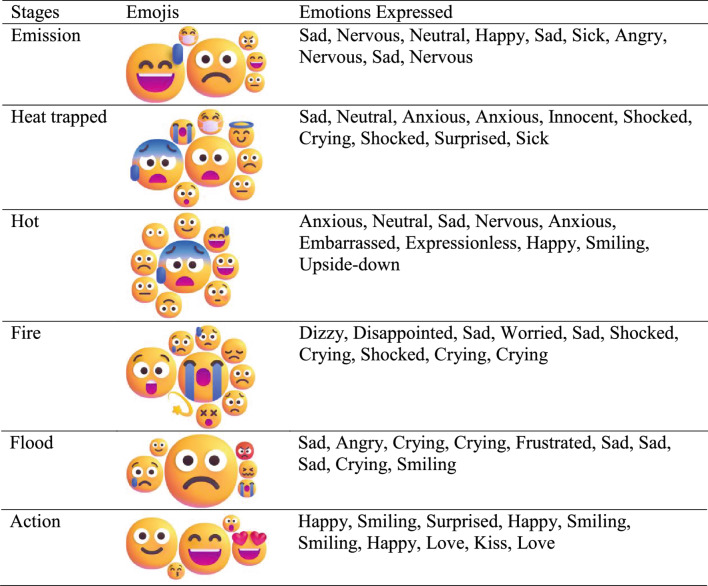
Findings from emoji-based reflection through gamification of the climate change process

### Identification of adaptation solutions self-identification and mapping of heatwave co-adaptation strategies

Table 5 summarizes students’ heatwave adaptation strategies, categorized into active and passive measures. Active strategies are defined as those requiring the direct consumption of resources or the operation of mechanical/electrical devices to alter the body’s immediate microclimate (e.g., using electric fans, drinking cold water, or utilizing air conditioning). Passive strategies are defined as behavioral or environmental adjustments that utilize existing infrastructure or spatial features to minimize heat gain (e.g., seeking shade, reducing physical exertion, or modifying clothing). Under this framework, ‘resting in a shaded area’ is classified as passive because the cooling effect is provided by the architectural or natural environment, regardless of the effort taken to reach the location.

**Table 5:** Summary of heatwave adaptation and preparedness solutions

No.	Proposed adaptation strategy	Frequency	Cooling type	Type of adaptation
1	Go to library for aircond	1	Active	Structural
2	Use proper attire	1	Passive	Behavioral
3	Rest in a shaded area	1	Passive	Behavioral
4	Drink more water	1	Active	Behavioral
5	Bring own handheld fan	2	Active	Technological
6	Sit under the fan	3	Active	Technological
7	Bring cold water from home	1	Active	Behavioral
8	Do not sit close together	1	Passive	Behavioral
9	Use hand fan	1	Active	Technological
10	Wash face	3	Active	Behavioral
11	Reduce outdoor activity	1	Passive	Behavioral
12	Use book as fan	2	Active	Behavioral
13	Go for aircond room	1	Active	Structural
14	Fold the shirt sleeves	1	Passive	Behavioral

Active and behavioral measures were most common, including sitting under fans and washing the face (reported by three groups), using handheld or book fans, visiting air-conditioned rooms, and drinking water. Passive and behavioral strategies, such as proper attire, resting in shaded areas, reducing outdoor activity, and folding shirt sleeves, were less frequent but important for minimizing heat gain. Technological adaptations (handheld fans, ceiling fans) complemented active interventions, while structural strategies (air-conditioned rooms, library access) reinforced thermal relief. Blue adaptations, such as drinking or bringing cold water, further supported physiological cooling. Overall, students combined immediate, low-cost actions with technological, environmental, and infrastructural adaptations, emphasizing active engagement, while passive, behavioral, blue, and structural strategies provided essential complementary relief. These findings align with global evidence on youth-driven heatwave adaptation, highlighting the importance of diverse strategies to address extreme heat in school environments (Kiarsi et al. 2023).

**Fig. 5:**
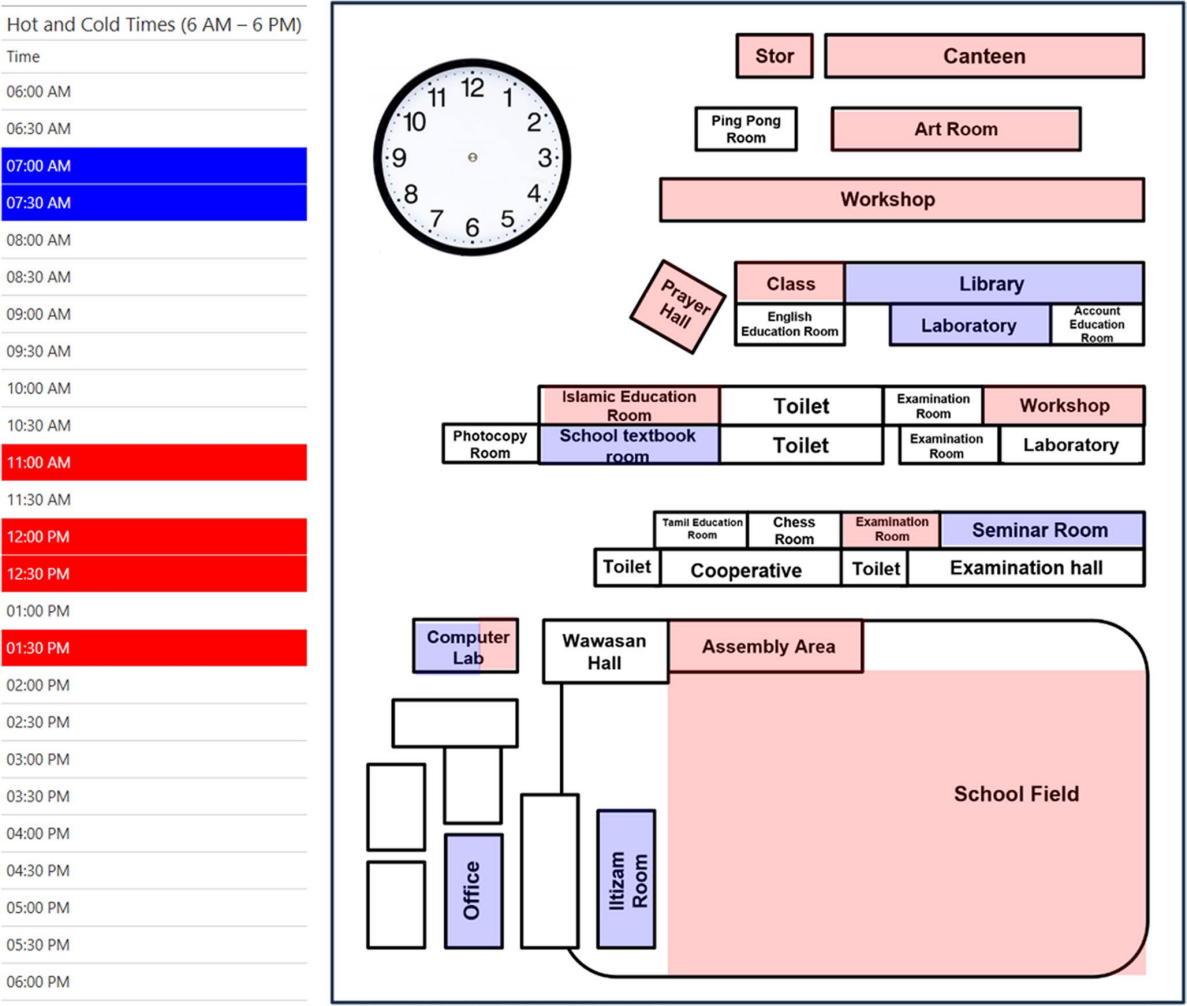
Identification of the school time and areal hotspot and cool spot based on dotmocracy co-adaptation mapping

Figure 5 shows that the coldest school period occurs between 7:00–7:30 AM, while the hottest period is 11:00 AM–1:30 PM. Morning arrival coincides with cooler conditions, but peak heat overlaps dismissal, exposing students to thermal stress during commuting and outdoor activities. Tropical classroom temperatures of ~ 28–34 °C can reduce comfort and concentration (Faraj et al. 2023), highlighting the need for shading, hydration, or activity adjustments. Spatially, cold spots were mainly indoor or shaded areas (libraries, labs, offices, seminar and textbook rooms, Iltizam room), whereas hot spots were in high-occupancy or sun-exposed spaces (workshops, canteens, prayer halls, classrooms, open fields). These patterns reveal a clear thermal gradient, consistent with Allah et al. (2023), where indoor/semi-enclosed spaces maintained 20–29 °C, while semi-outdoor and outdoor areas reached up to 29 °C, emphasizing targeted adaptation strategies within school environments. The details mapping during the exercise can be found in Supplementary Material 7.

The heatwave co-adaptation mapping by Groups 1–6 identified thermally stressed hotspots across the school, including the workshop, canteen, field, computer lab, art room, prayer hall, store, and Islamic education room, revealing site-specific thermal challenges shaped by structural design, function, and occupancy. Workshops were among the hottest indoor spaces due to direct solar exposure, limited ventilation, and high occupancy, consistent with Mat Alias and Kassim (2023), where 66.5% of Malaysian TVET workshop users reported discomfort (average TSV = 1.85). Proposed adaptations included air-conditioning installation, vent addition, and room layout reorganization, reflecting combined engineering and bioclimatic approaches.

The canteen faced poor air circulation, high traffic, and dust accumulation, with solutions emphasizing energy-efficient fans, additional ventilation openings, scheduled fan use, and maintenance to improve thermal and air quality. The school field was the most heat-exposed outdoor zone; adaptations included shade trees, canopies, shaded activity areas, bicycle-powered water sprinklers, fish ponds, and hydration stations, supporting physiological and ecological adaptation. These align with green-blue-grey strategies exemplified by the Barcelona “Climate Shelters in Schools” project, which installed 74 trees and 26 water points across 11 schools (Sanz-Mas et al. 2024). Computer labs suffered heat from devices and inefficient cooling; proposed measures included fan supplementation, equipment reorganization, and regular maintenance. Indoor learning spaces—art room, examination room, Islamic education room—required ventilation improvements and air-conditioning upgrades, with vegetation added for shading and evapotranspiration. Prayer halls and stores similarly benefited from ventilation enhancement and passive cooling. Firman et al. (2025) reported that ~ 75% of Malaysian secondary students experienced thermal comfort around 29.3 °C, supporting the critical role of these interventions.

Behavioral adaptations complemented physical measures across all hotspots, including lighter clothing, fan management, hydration, and gradual acclimatization, consistent with Tablada et al. (2022), who reported that students adapt behaviorally even when classrooms exceed thermal comfort ranges. Collectively, the findings demonstrate that differentiated, multi-scalar interventions, such as mechanical, structural, green, and behavioral, co-created by students enhance thermal comfort and resilience, emphasizing integrated technical, ecological, and behavioral strategies for effective school-based heatwave adaptation. The heatwave co-adaptation mapping exercises conducted by Groups 1 to 6 collectively provided a comprehensive understanding of thermally stressed zones across various school environments and proposed context-specific adaptation strategies that integrate various measures to reduce indoor heat stress, and to promote adaptive capacity among students and staff during extreme heat conditions. Several key hotspots were revealed across the school compound, including the workshop, canteen, school field, computer lab, art room, prayer hall, store, and Islamic education room. Each location presented unique thermal challenges shaped by its structural characteristics, functional use, and occupancy patterns, necessitating targeted adaptation responses that integrate infrastructural, behavioral, and nature-based measures.

### Type of adaptation: an alluvial plot analysis

**Fig. 6:**
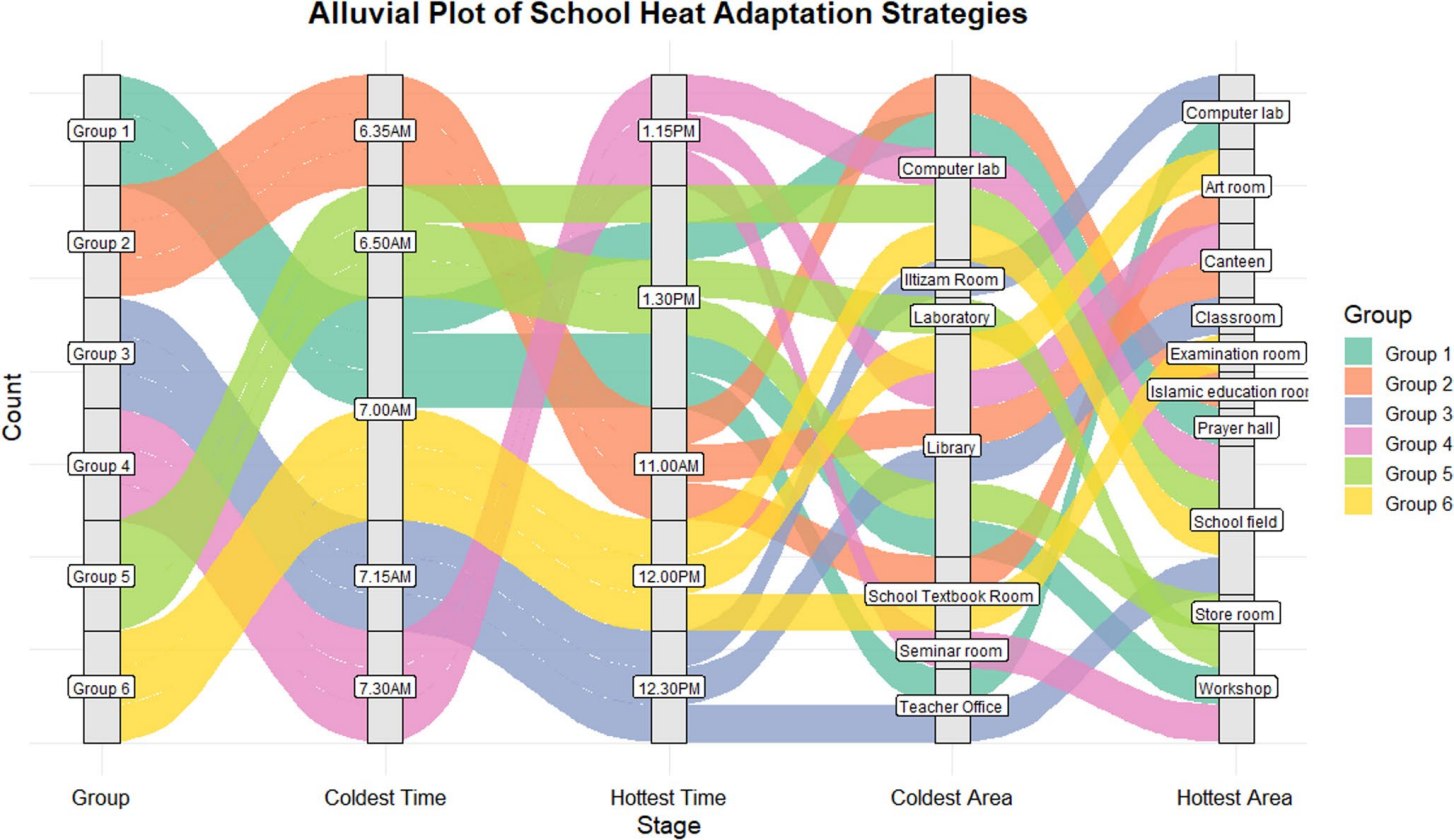
Alluvial plot showing the flow of school heatwave co-adaptation strategies across six groups. Each group is assigned a distinct color, and the stratum labels indicate the specific categories at each stage

The alluvial plot in Fig. 6 reveals clear patterns in the distribution of heatwave co-adaptation strategies across different groups. Each flow illustrates how specific groups experience variations in thermal conditions, from the coldest to hottest times and areas. Notably, certain areas such as the library and computer lab repeatedly appear as cold spots, while workshops and canteens emerge consistently as hot spots, suggesting localized thermal vulnerabilities. The visualization of transitions between time and space emphasizes that adaptation strategies are not uniformly applied. Instead, they are group-specific and influenced by students’ preferences, with certain types of students tending toward particular adaptation strategies. The detail of the preferred adaptation strategies was given in Supplementary Material 8, categorized as behavioral, blue, design, ecological, green, health-adaptive, institutional, maintenance, nature-based, passive, physiological, psychological, renewable, structural, and technological.

In was observed that the groups that identified the same cold or hot spots often proposed both similar and differing adaptation strategies. For instance, Groups 1 and 2 both recognized the library as a cold spot. However, Group 1 focused on structural and technological solutions (installing air-conditioning, additional fans, optimizing room layout), whereas Group 2 combined structural measures with maintenance and institutional strategies (fan schedules, room closures, cleaning routines), showing variation in approach despite shared spatial perception. Similarly, for hot spots such as the workshop and canteen, Groups 4 and 5 both prioritized structural interventions, but Group 4 incorporated blue (water-based) and renewable adaptations like small fountains or misting systems, while Group 5 emphasized behavioral and green strategies (shade structures, vegetation, short-sleeved uniforms). This comparison highlights that even when students identify the same thermal risk areas, their choice of adaptation strategies reflects differing priorities, experiences, and perceived control.

For example, Groups 1 and 2 prioritized structural and technological interventions, reflecting a preference for infrastructural and design-based solutions. Groups 3 and 5 emphasized nature-based and green strategies, indicating that students with higher environmental awareness or familiarity with outdoor spaces favored ecological and behavioral approaches. Group 6 highlighted blue and health-adaptive strategies, such as hydration stations and encouraging cooling practices (drink water, wash face, sit under fans), suggesting a focus on physiological comfort and health-conscious behaviors. The groups highlight differences in exposure and potential behavioral responses, providing information into prioritizing interventions. This information is crucial in facilitating the identification of critical areas and periods where heat adaptation measures could be targeted effectively. Overall, the plot underscores the need for tailored adaptation strategies that consider both temporal and spatial heterogeneity in school environments.

In term of the type of adaptation, structural and technological adaptations dominated, appearing in > 70% of strategies. Structural measures included insulation, shaded roofing, ventilation, and heat-reflective materials, while technological measures encompassed air-conditioning, fans, sensors, and renewable energy systems, reflecting infrastructure-based primary defenses against school heat stress (Wu et al. 2025). Design adaptations, optimizing airflow, orientation, and layout, were frequently paired with structural strategies, indicating a passive cooling focus. Behavioral and psychological adaptations appeared in ~ 20–25% of strategies, including hydration, activity adjustment, and heat-awareness training, emphasizing the human dimension of adaptation (Riaz et al. 2025). Institutional measures (~ 15%) involved school heat protocols, early-warning dissemination, and teacher-led planning, highlighting governance integration Lao et al. (2025). Maintenance, though less frequent, addressed regular servicing of cooling systems to ensure long-term functionality.

Nature-based, green, and blue adaptations (~ 30%) promoted ecosystem-based resilience through vegetation, water features, rooftop gardens, and hydration stations, enhancing thermal comfort, biodiversity, and psychological well-being. Renewable adaptations, though rare, included solar panels to sustainably power cooling systems. Passive and health-adaptive measures emphasized natural ventilation, heat dispersion, hydration, and heat exposure management. Overall, a hierarchical pattern emerged: structural-technological solutions were dominant, followed by ecological and behavioral strategies, with institutional, psychological, and maintenance measures supporting long-term resilience. The comparison of strategies across groups that shared the same cold or hot spots indicates that students’ adaptation choices are influenced not only by exposure but also by perceived control, familiarity with the environment, and comfort priorities, reinforcing the need to account for student characteristics when planning school heatwave interventions. This aligns with Das et al. (2025), which found building modifications most frequent, with landscape/green and behavioral measures less common. Effective school heatwave adaptation requires integrating physical infrastructure, ecological interventions, and human behavior into a synergistic, multi-layered system.

## Conclusion

This study presents a detailed assessment of heatwave exposure and co-adaptation strategies within school environments, integrating data-driven heatwave assessment, hotspot mapping, and participatory adaptation exercises. The findings highlight significant temporal and spatial variations in heat stress, with indoor hotspots including workshops, computer labs, canteens, art rooms, and prayer halls, and outdoor hotspots primarily in school fields exposed to direct sunlight. Students proposed a diverse set of context-specific adaptations encompassing structural, technological, design, behavioral, ecological, green, blue, passive, health-adaptive, and institutional measures. Structural and technological interventions emerged as the most prevalent (> 70%), complemented by nature-based (~ 30%) and behavioral (~ 20–25%) strategies, demonstrating a multi-layered, multi-scalar approach to enhancing thermal comfort and resilience. Behavioral and psychological adaptations emphasized the human dimension of heatwave response, while institutional and maintenance measures underscored the importance of governance and long-term sustainability. The co-adaptation exercises also revealed the potential of student engagement and participatory methods in promoting climate literacy, exploring and improving adaptive capacity with practical solution-oriented thinking.

Limitations of this study include its focus on a single school context, which may limit the generalizability of findings to other school types or climatic settings. Participant selection was constrained to a single age group and selected participants, potentially introducing selection bias and limiting representativeness of the broader youth population in Segamat or Malaysia. Additionally, the adaptation proposals are largely theoretical, derived from perceptions and group exercises rather than longitudinal implementation and effectiveness testing. Thermal monitoring was constrained to specific periods, and dynamic factors such as seasonal variability, occupancy changes, and real-time weather fluctuations were not fully captured. The small sample size and overrepresentation of certain racial groups (e.g., Malay students) may also influence findings and limit extrapolation. Future work should involve longitudinal implementation of the proposed adaptations to evaluate their effectiveness in real-world conditions, coupled with continuous thermal and physiological monitoring. Expanding the study to multiple schools across diverse climatic regions would strengthen the generalizability of findings. Integration of advanced modeling techniques, including microclimate simulation and energy-efficient building design, alongside participatory frameworks, can optimize adaptation strategies. Finally, linking adaptation measures with educational outcomes and student well-being would provide holistic insights into the co-benefits of school-based heatwave resilience interventions.

## Supplementary Information

Below is the link to the electronic supplementary material.

Supplementary File 1 (DOCX 2.14 MB)

## Data Availability

Not applicable.
