# Supplementary material for: Youth-led spatio-temporal heatwave co-adaptation mapping through data-driven participatory dotmocracy approach
Source: Int J Biometeorol. 2026 May 8;70(5):157. doi: 10.1007/s00484-026-03218-0 (PMC13156233; doi:10.1007/s00484-026-03218-0)
Supplement: Supplementary file 1 — (DOCX 2.14 MB) [file 484_2026_3218_MOESM1_ESM.docx]

Supplementary material 1. Pre-survey assessment (Translated from Malay to English).

**CLIMATE AMBASSADORS PROGRAM SURVEY FORM**

**(Before)**

**Instructions**: Please answer all questions honestly.

## Section A: Basic Information

Name : _______________________________________________

Gender : _______________________________________________

Form : _______________________________________________

Have you previously participated in any environmental-related program?

☐ Yes ☐ No

## Section B: Basic Knowledge (Select one answer only)

1. What is the difference between weather and climate?

☐ A. There is no difference

☐ B. Weather is daily, climate is a long-term pattern

☐ C. Climate depends on school holiday seasons

1. Which of the following is NOT an effect of climate change?

☐ A. Rising temperatures

☐ B. Flash floods

☐ C. Slow internet

1. What does “carbon footprint” mean?

☐ A. A footprint in the mud

☐ B. The amount of greenhouse gases from human activities

☐ C. The name of an eco-friendly shoe company

1. Which of the following actions helps reduce climate impacts?

☐ A. Burning trash

☐ B. Turning off lights when not in use

☐ C. Leaving the tap running

1. Which of the following is NOT an impact of climate change on water resources?

☐ A. The quantity and quality of water resources such as rivers may be affected

☐ B. Flooding

☐ C. River water becomes cooler

Section C: Level of Awareness and Attitude

Please indicate your level of agreement for each of the following statements.

(1 = Strongly disagree, 5 = Strongly agree)

|  |  | **1** |  | **2** |  | **3** |  | **4** |  | **5** |
| --- | --- | --- | --- | --- | --- | --- | --- | --- | --- | --- |
| 1. | I am aware that my actions can impact the environment and climate change. |  |  |  |  |  |  |  |  |  |
| 2. | I understand that climate change can affect the quality and quantity of water. |  |  |  |  |  |  |  |  |  |
| 3. | I will save electricity to reduce the risks of climate change. |  |  |  |  |  |  |  |  |  |
| 4. | I am aware of the importance of heatwave risk mapping in addressing climate change. |  |  |  |  |  |  |  |  |  |
| 5. | I am confident that applying knowledge in STEM can help reduce the risks of climate change. |  |  |  |  |  |  |  |  |  |
| 6. | I am interested in exploring STEM fields to solve environmental problems. |  |  |  |  |  |  |  |  |  |
| 7. | I believe I can become an agent of change in my school or community. |  |  |  |  |  |  |  |  |  |

## Section D: Short Open-Ended Responses

1. In one sentence, what do you understand about climate change?

2. What is one thing you can change to help the environment?

3. What is your suggestion to make the school more environmentally friendly?

Supplementary material 2. Post-survey assessment (Translated from Malay to English).

**CLIMATE AMBASSADORS PROGRAM SURVEY FORM**

**(After)**

**Instructions**: Please answer all questions honestly.

## Section A: Basic Information

Name : _______________________________________________

Gender : _______________________________________________

Form : _______________________________________________

Pernahkah anda menyertai program berkaitan alam sekitar sebelum ini?

☐ Ya ☐ Tidak

## Section B: Basic Knowledge (Select one answer only)

1. What is the difference between weather and climate?

☐ A. There is no difference

☐ B. Weather is daily, climate is a long-term pattern

☐ C. Climate depends on school holiday seasons

1. Which of the following is NOT an effect of climate change?

☐ A. Rising temperatures

☐ B. Flash floods

☐ C. Slow internet

1. What does “carbon footprint” mean?

☐ A. A footprint in the mud

☐ B. The amount of greenhouse gases from human activities

☐ C. The name of an eco-friendly shoe company

1. Which of the following actions helps reduce climate impacts?

☐ A. Burning trash

☐ B. Turning off lights when not in use

☐ C. Leaving the tap running

1. Which of the following is NOT an impact of climate change on water resources?

☐ A. The quantity and quality of water resources such as rivers may be affected

☐ B. Flooding

☐ C. River water becomes cooler

Section C: Level of Awareness and Attitude

Please indicate your level of agreement for each of the following statements.

(1 = Strongly disagree, 5 = Strongly agree)

|  |  | **1** |  | **2** |  | **3** |  | **4** |  | **5** |
| --- | --- | --- | --- | --- | --- | --- | --- | --- | --- | --- |
| 1. | I am aware that my actions can impact the environment and climate change. |  |  |  |  |  |  |  |  |  |
| 2. | I understand that climate change can affect the quality and quantity of water. |  |  |  |  |  |  |  |  |  |
| 3. | I will save electricity to reduce the risks of climate change. |  |  |  |  |  |  |  |  |  |
| 4. | I am aware of the importance of heatwave risk mapping in addressing climate change. |  |  |  |  |  |  |  |  |  |
| 5. | I am confident that applying knowledge in STEM can help reduce the risks of climate change. |  |  |  |  |  |  |  |  |  |
| 6. | I am interested in exploring STEM fields to solve environmental problems. |  |  |  |  |  |  |  |  |  |
| 7. | I believe I can become an agent of change in my school or community. |  |  |  |  |  |  |  |  |  |

## Section D: Short Open-Ended Responses

1. In one sentence, what do you understand about climate change?

2. What is one thing you can change to help the environment?

3. What is your suggestion to make the school more environmentally friendly?

Supplementary material 3. Tri-brochure “Reflection: My Climate Voice”. (Translated from Malay to English)
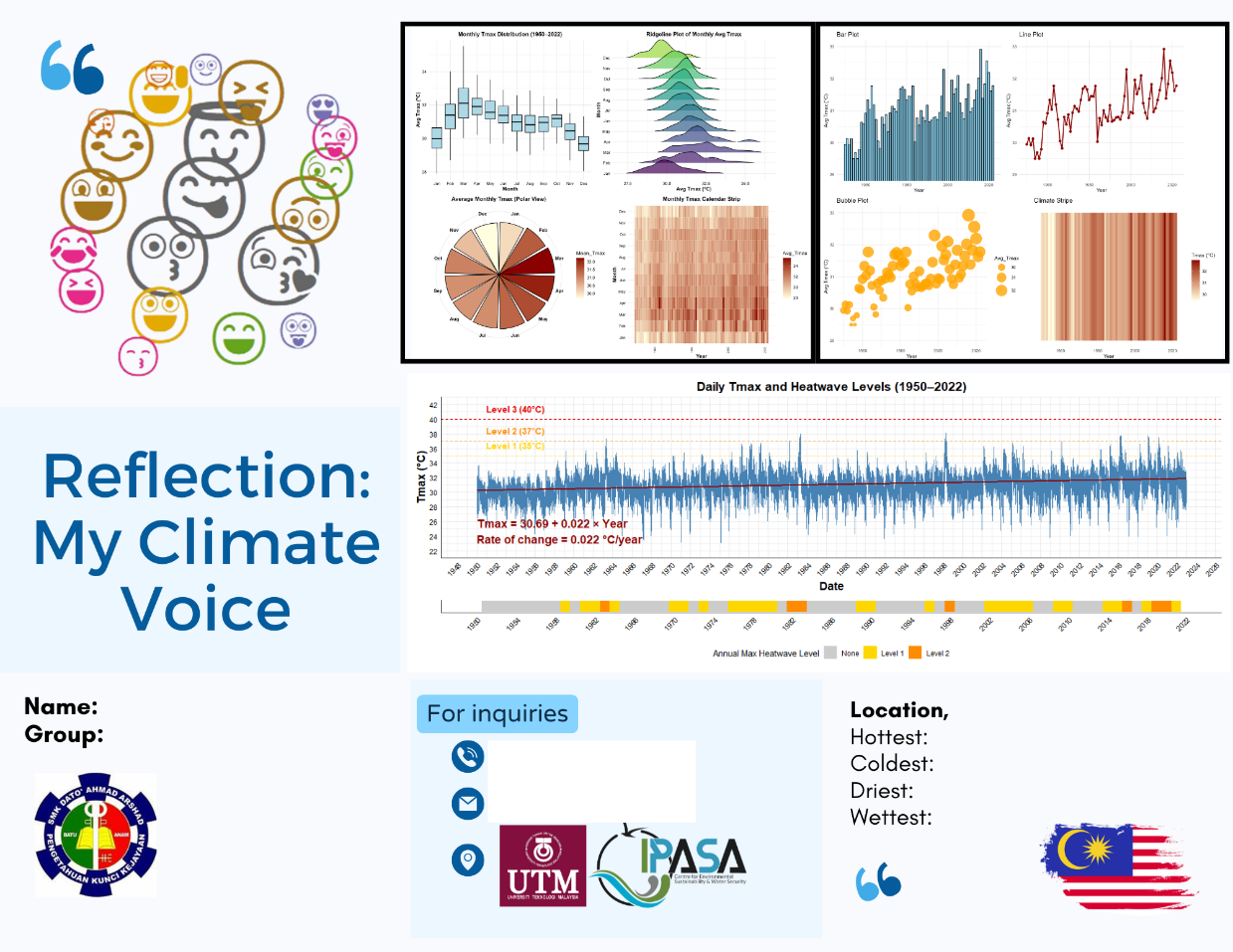


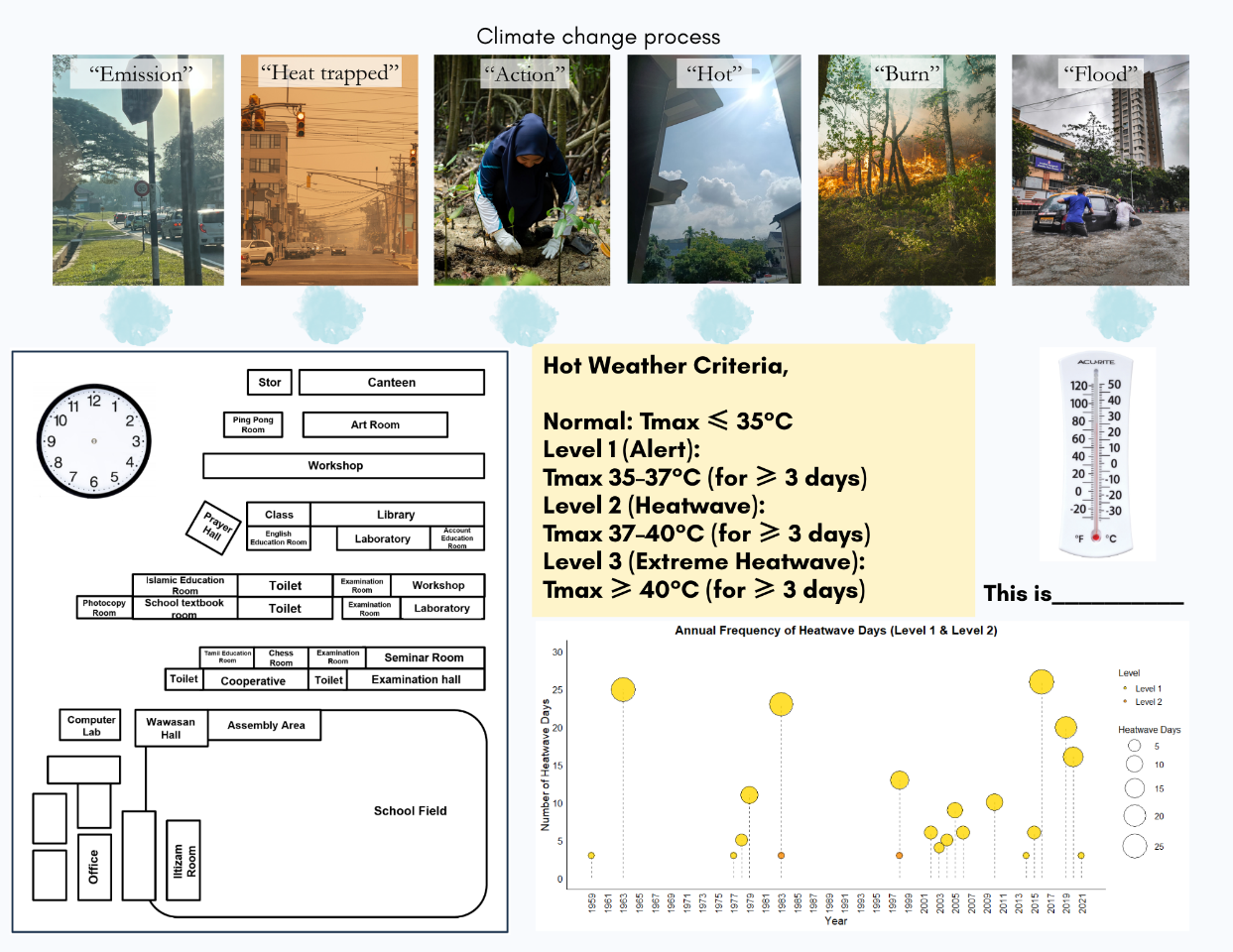


Supplementary material 4. Findings from emoji-based reflection through gamification of climate change process. (Translated from Malay to English)


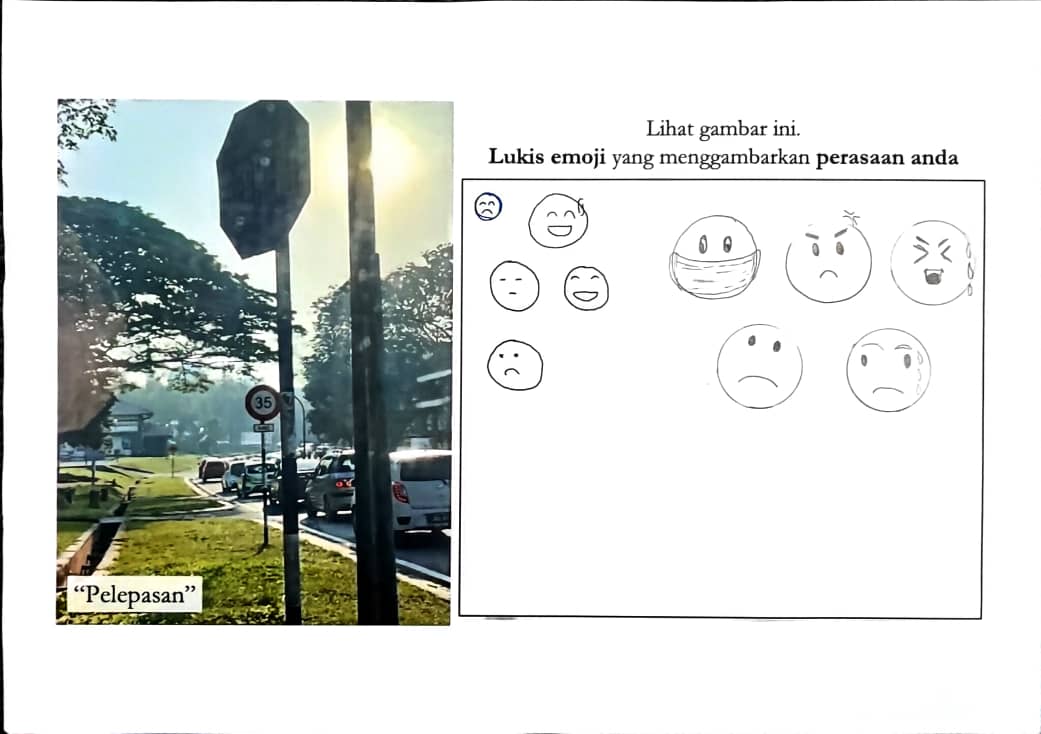

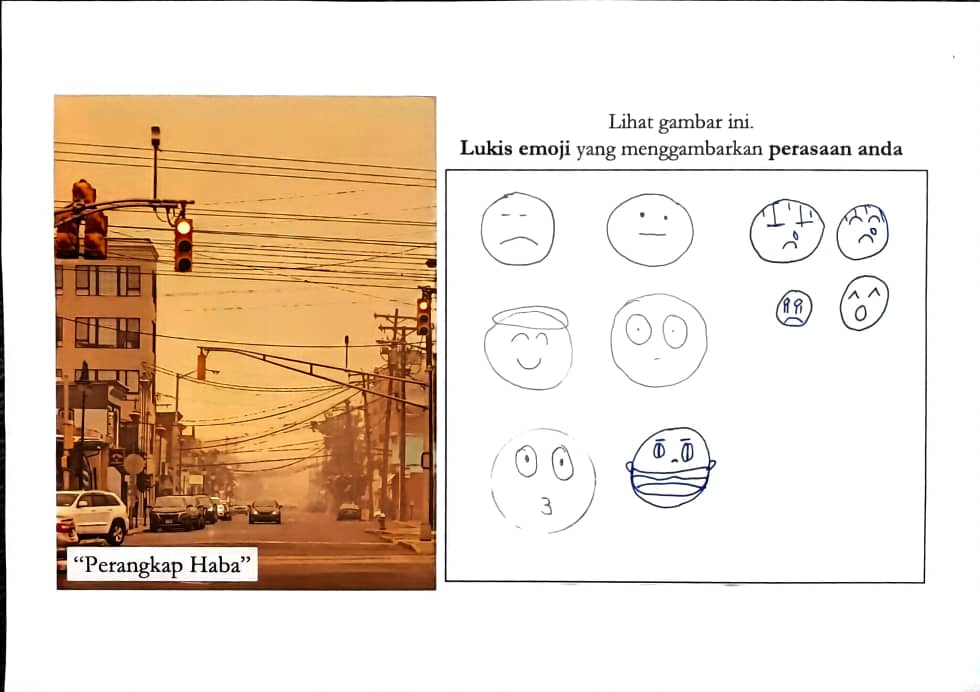


“Heat trapped”

“Emission”

Look at this picture.
Draw an emoji that represents how you feel.

Look at this picture.
Draw an emoji that represents how you feel.


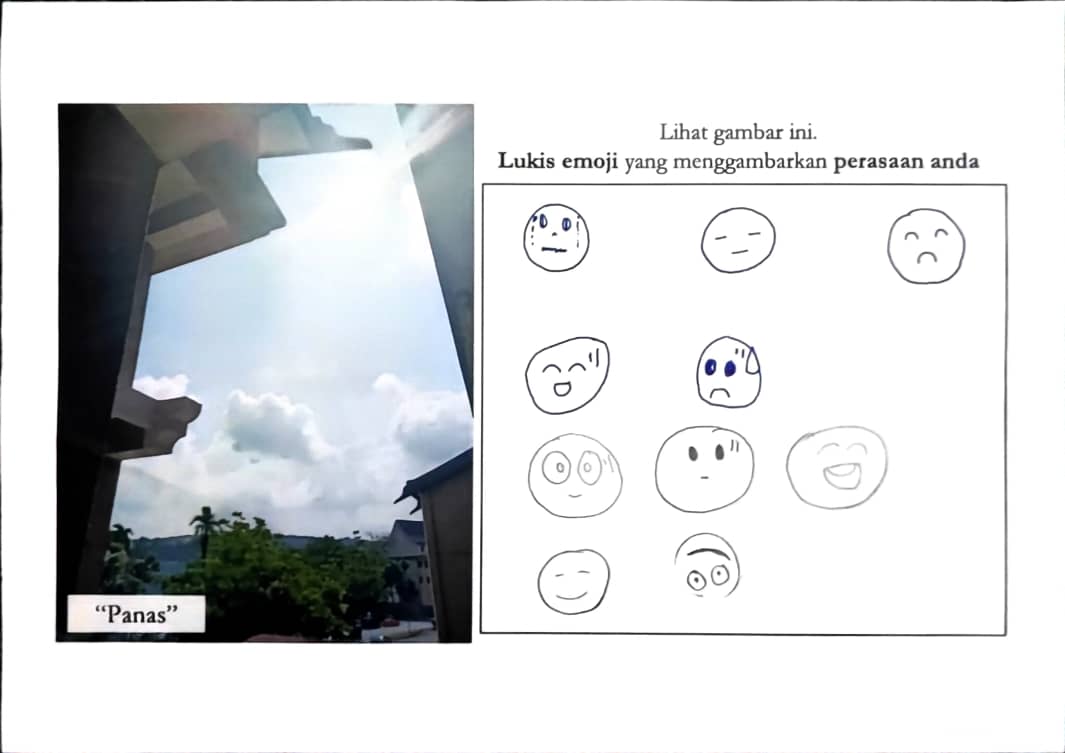

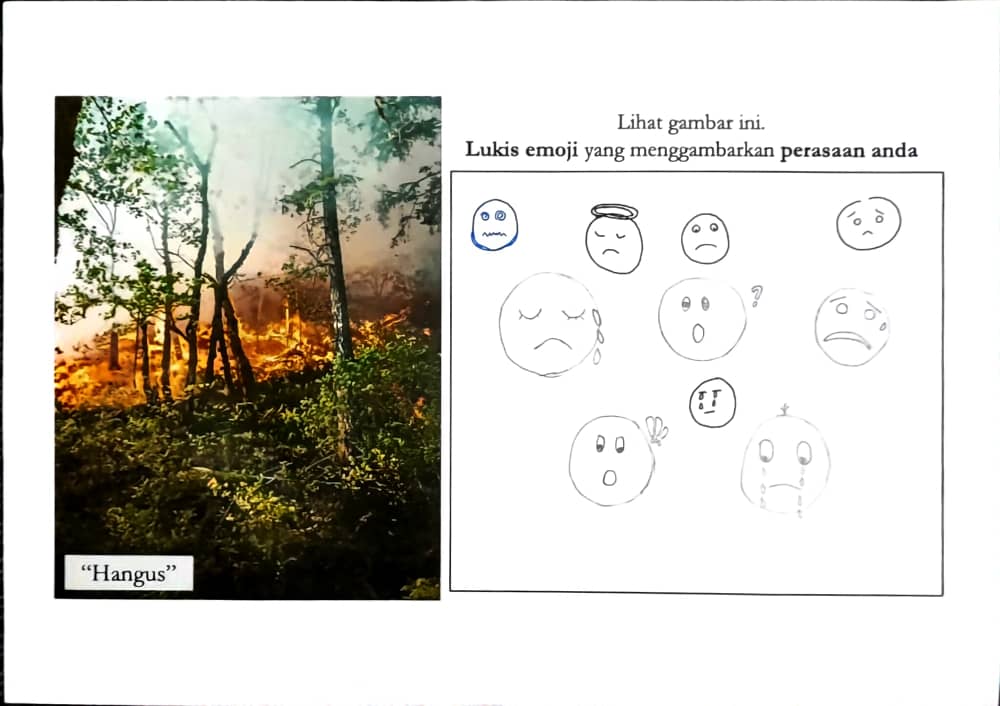

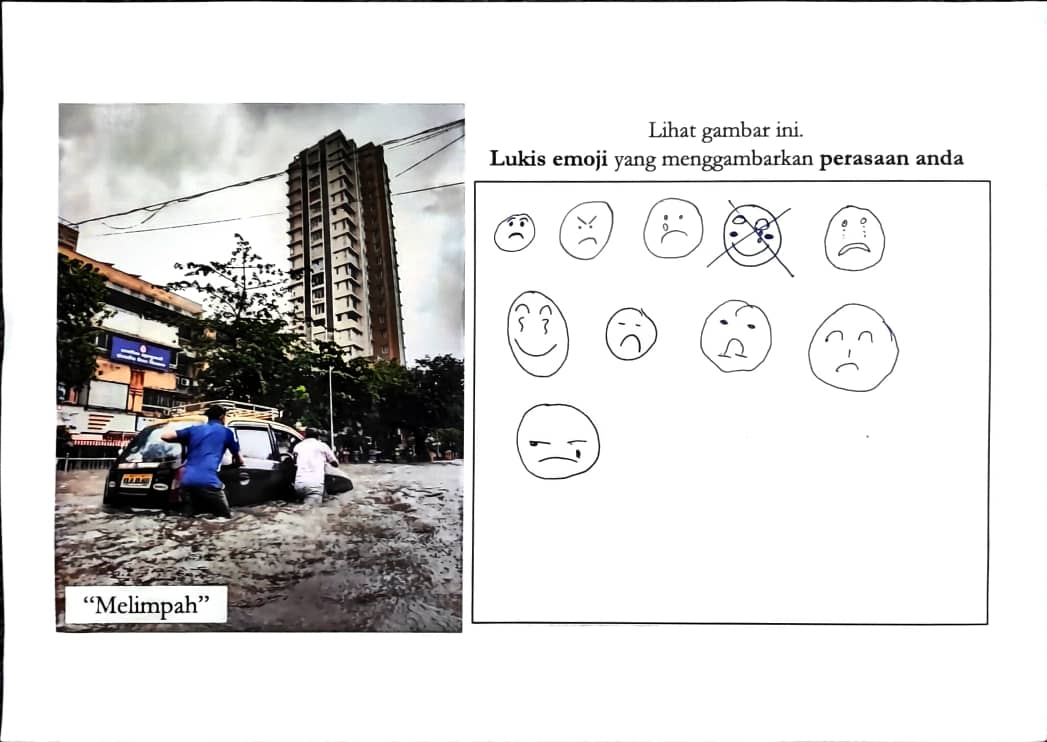

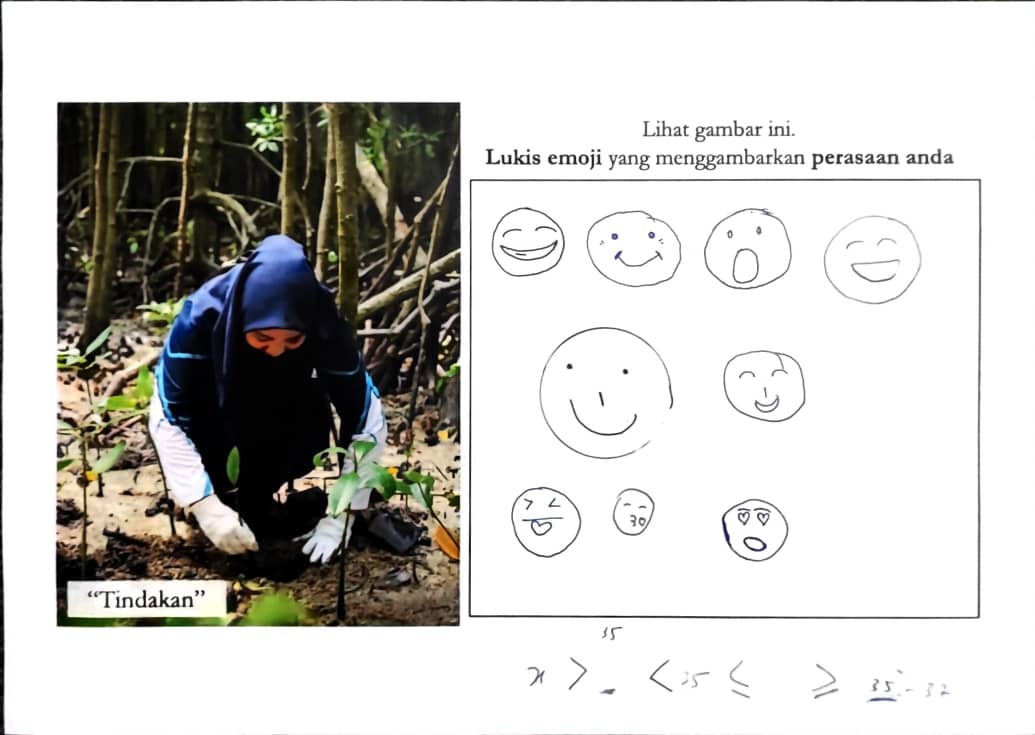


“Burn”

“Hot”

Look at this picture.
Draw an emoji that represents how you feel.

Look at this picture.
Draw an emoji that represents how you feel.

“Flood”

Look at this picture.
Draw an emoji that represents how you feel.

“Action”

Look at this picture.
Draw an emoji that represents how you feel.

Supplementary material 5. (a) Distribution of heatwave intensities, and (b) boxplot of heatwave intensity, respectively, by severity level (Level 1 in yellow and Level 2 in orange).


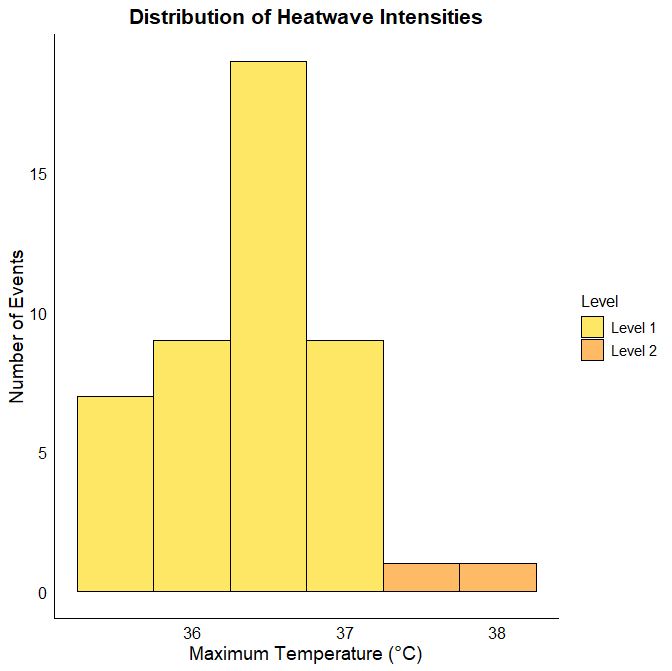

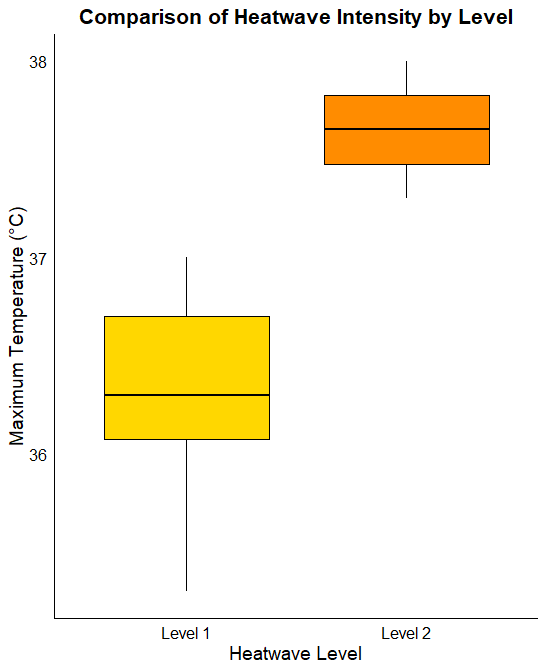


(a)

(b)

Supplementary Material 6. Results of McNemar’s test with Bonferroni adjustment assessing changes in knowledge before and after the intervention for Q1-Q5. Percentages of correct responses before and after the intervention are shown for each question, along with the original p-values, Bonferroni-adjusted p-values, and significance change. The questions in Malay were translated to English.

| Question | Correct Before (%) | Correct After (%) | p-value | Significance | Bonferroni | Significant |
| --- | --- | --- | --- | --- | --- | --- |
| B1. What is the difference between weather and climate? | 72.41 | 96.55 | 0.046 | ✔ | 0.228 | No |
| B2. Which of the following is NOT an effect of climate change? | 65.52 | 93.1 | 0.027 | ✔ | 0.135 | No |
| B3. What is the meaning of ‘carbon footprint’? | 89.66 | 93.1 | 1.000 | ✘ | 1.000 | No |
| B4. Which of the following actions helps reduce the impacts of climate change? | 68.97 | 93.1 | 0.023 | ✔ | 0.117 | No |
| B5. Which of the following is NOT an effect of climate change on water resources? | 51.72 | 65.52 | 0.386 | ✘ | 1.000 | No |

Supplementary material 7. Co-adaptation mapping by each group.


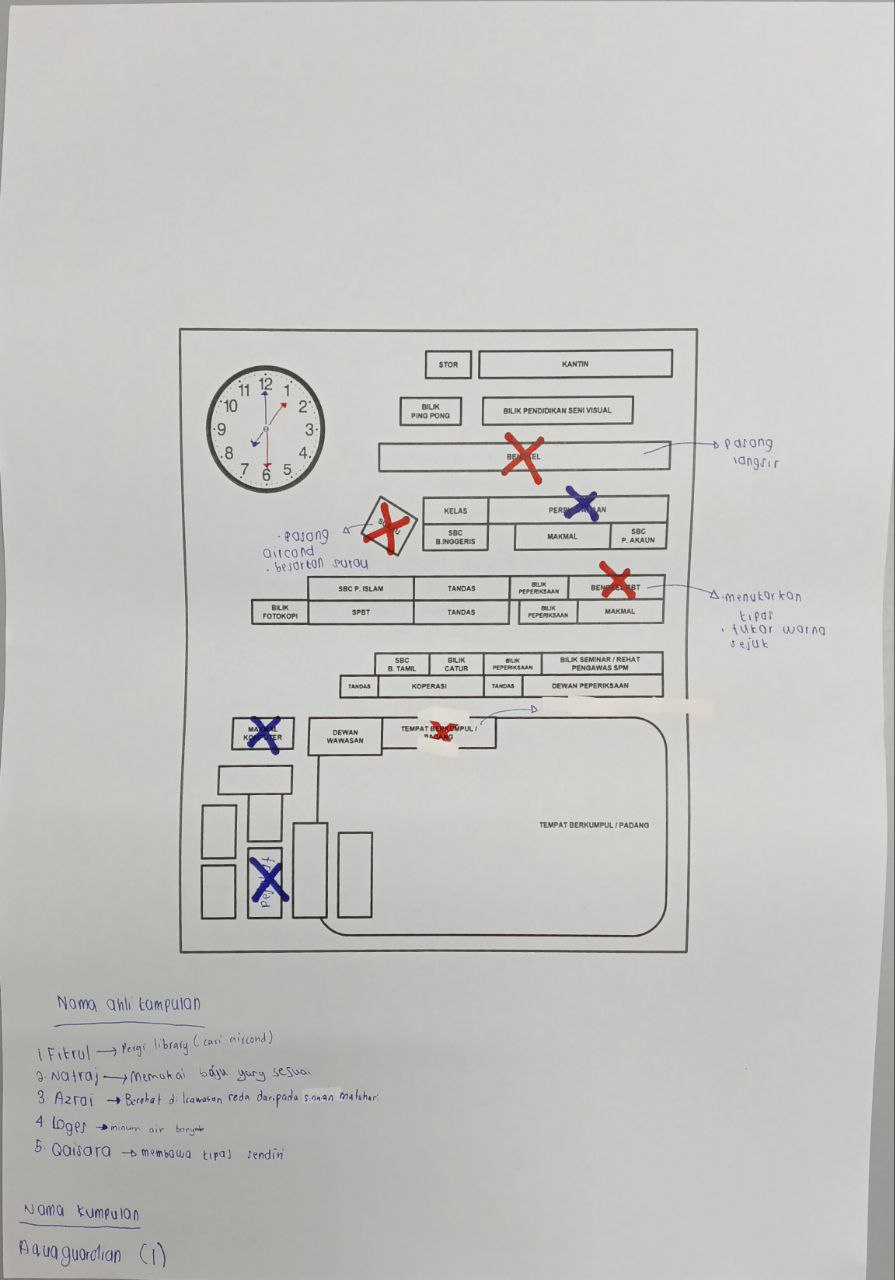


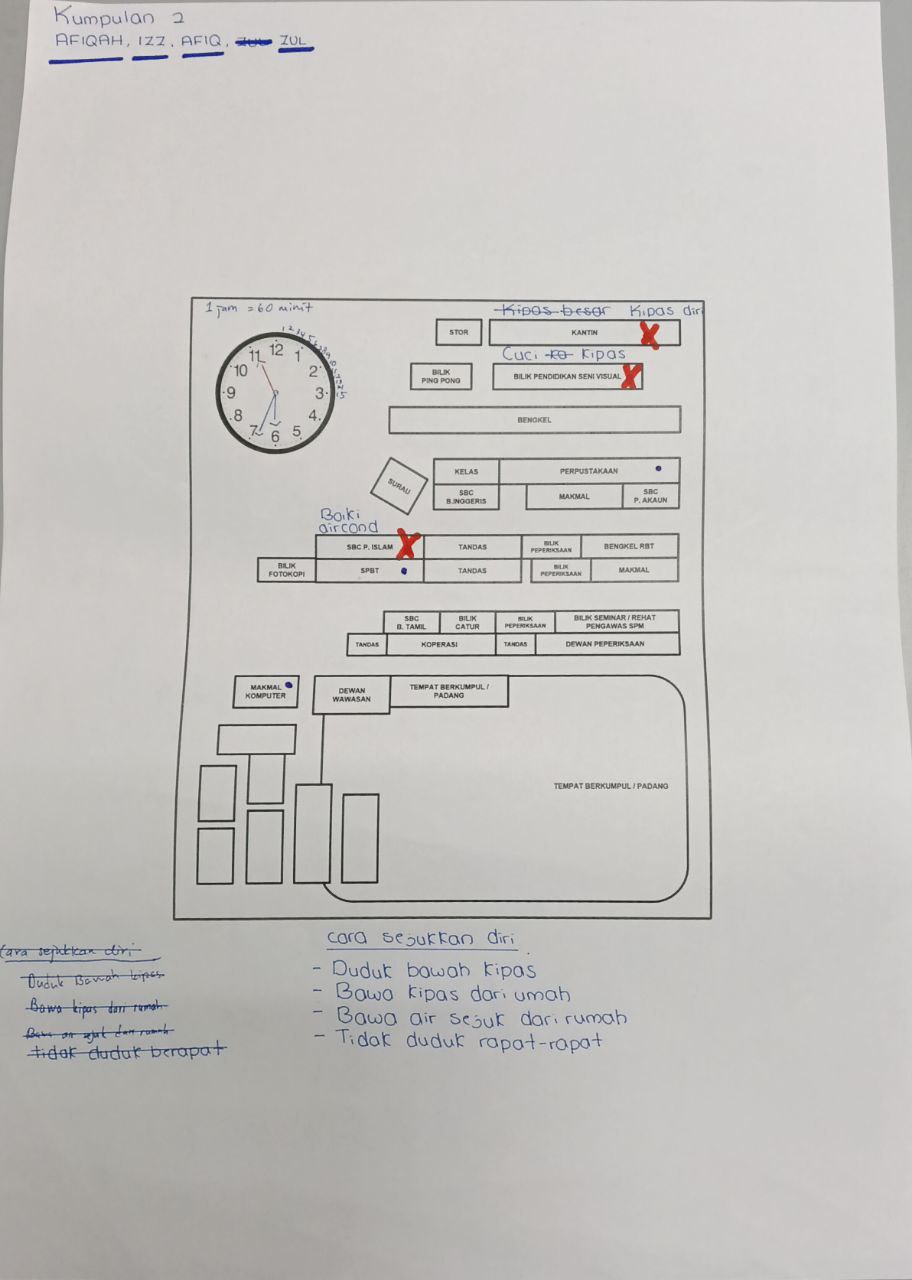


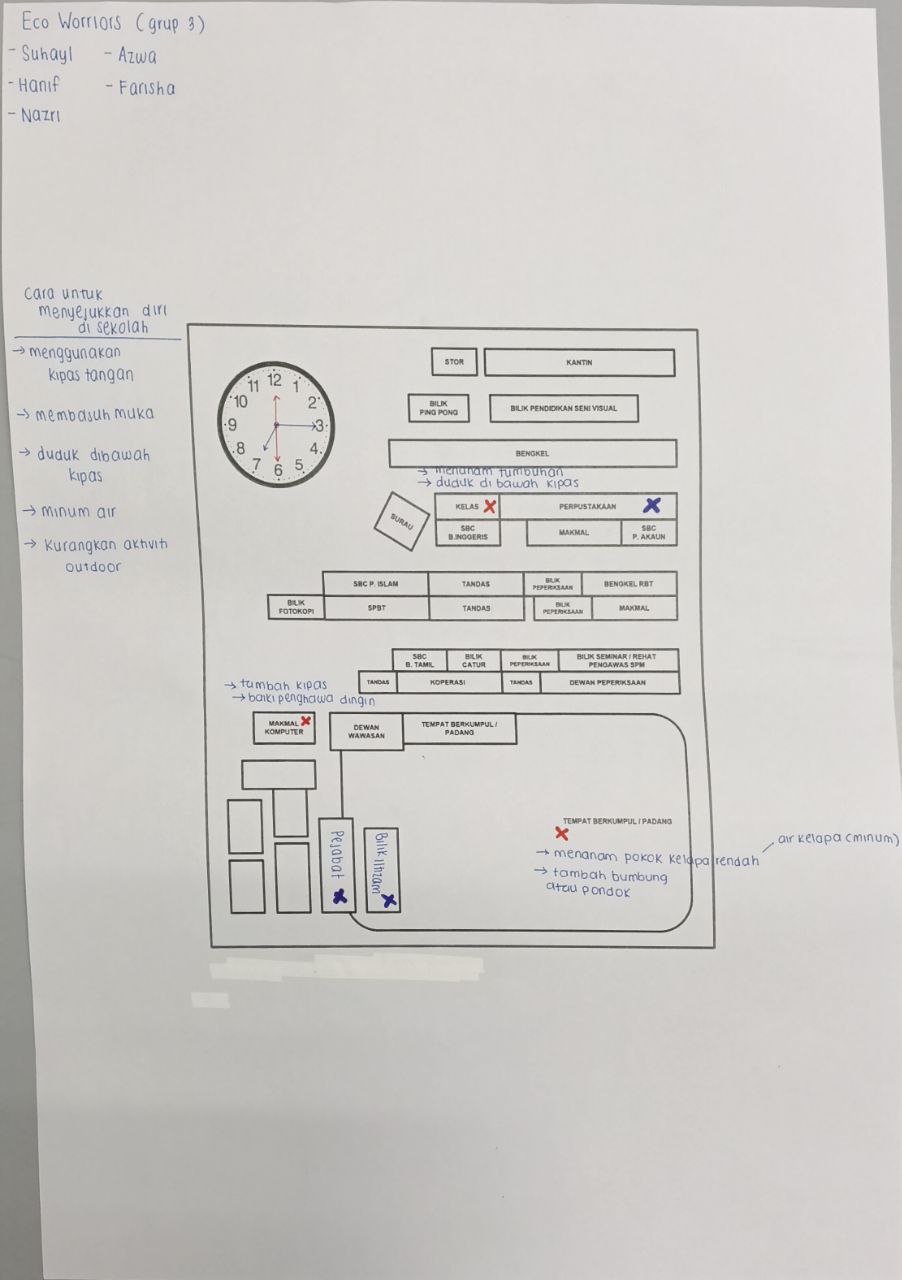


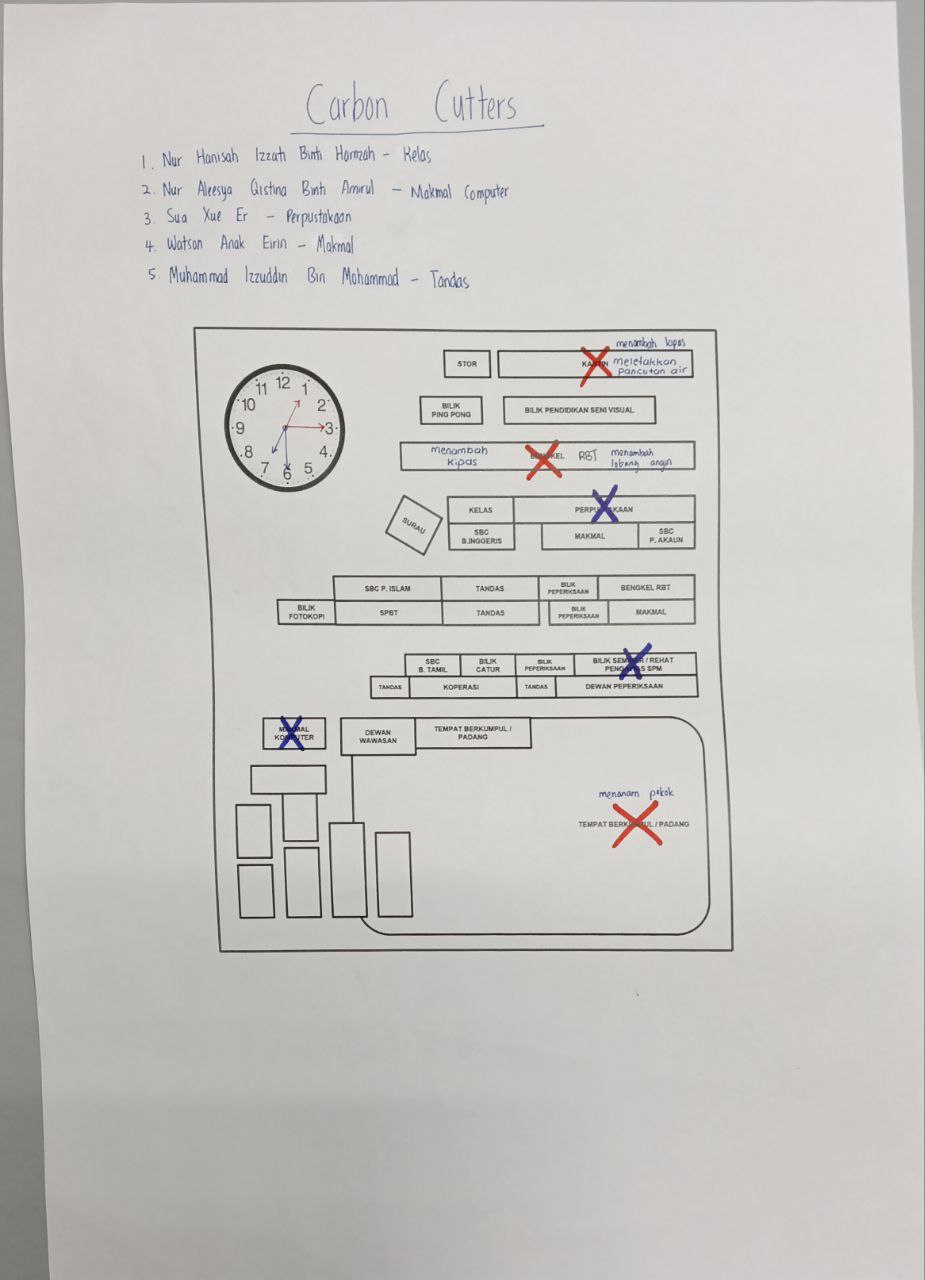


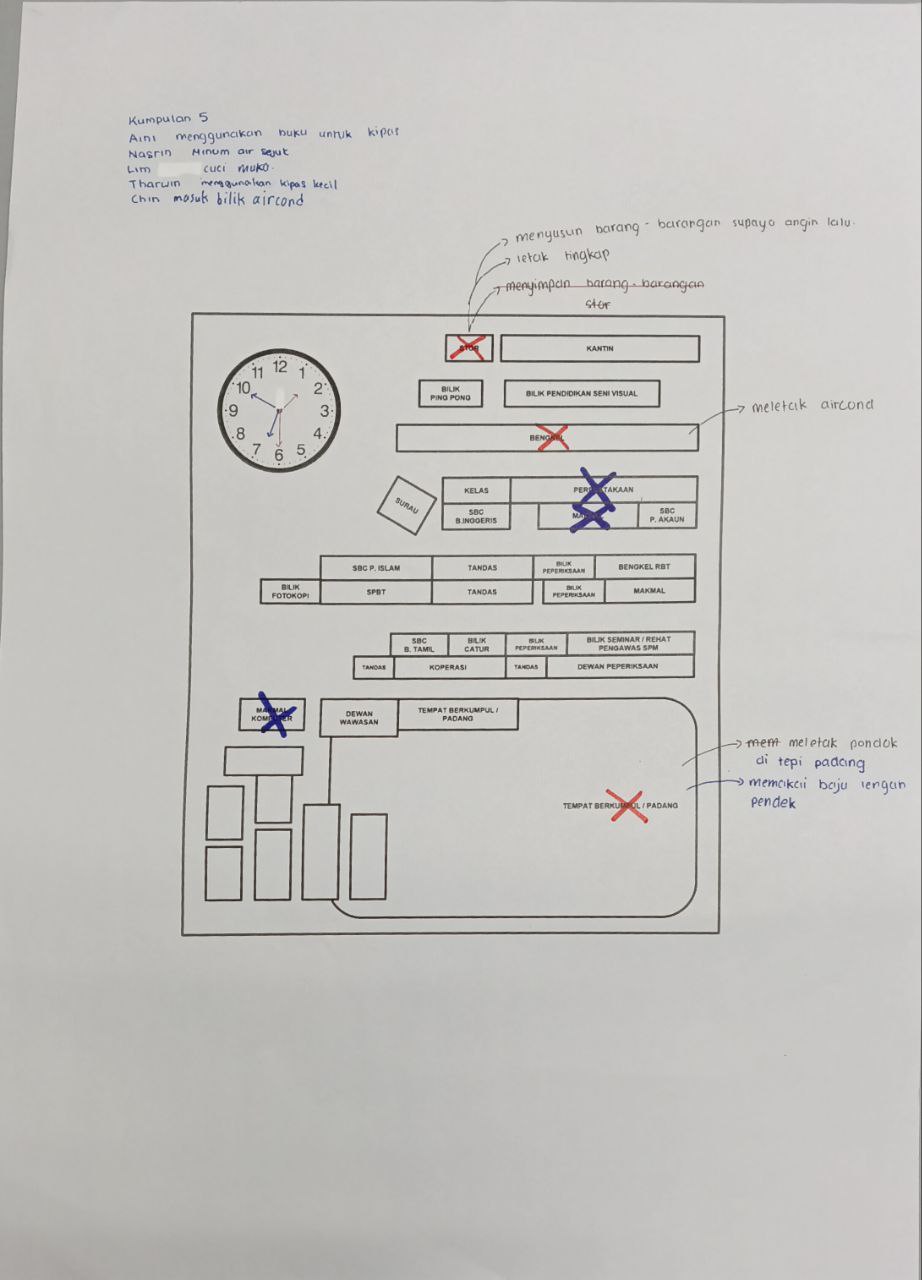


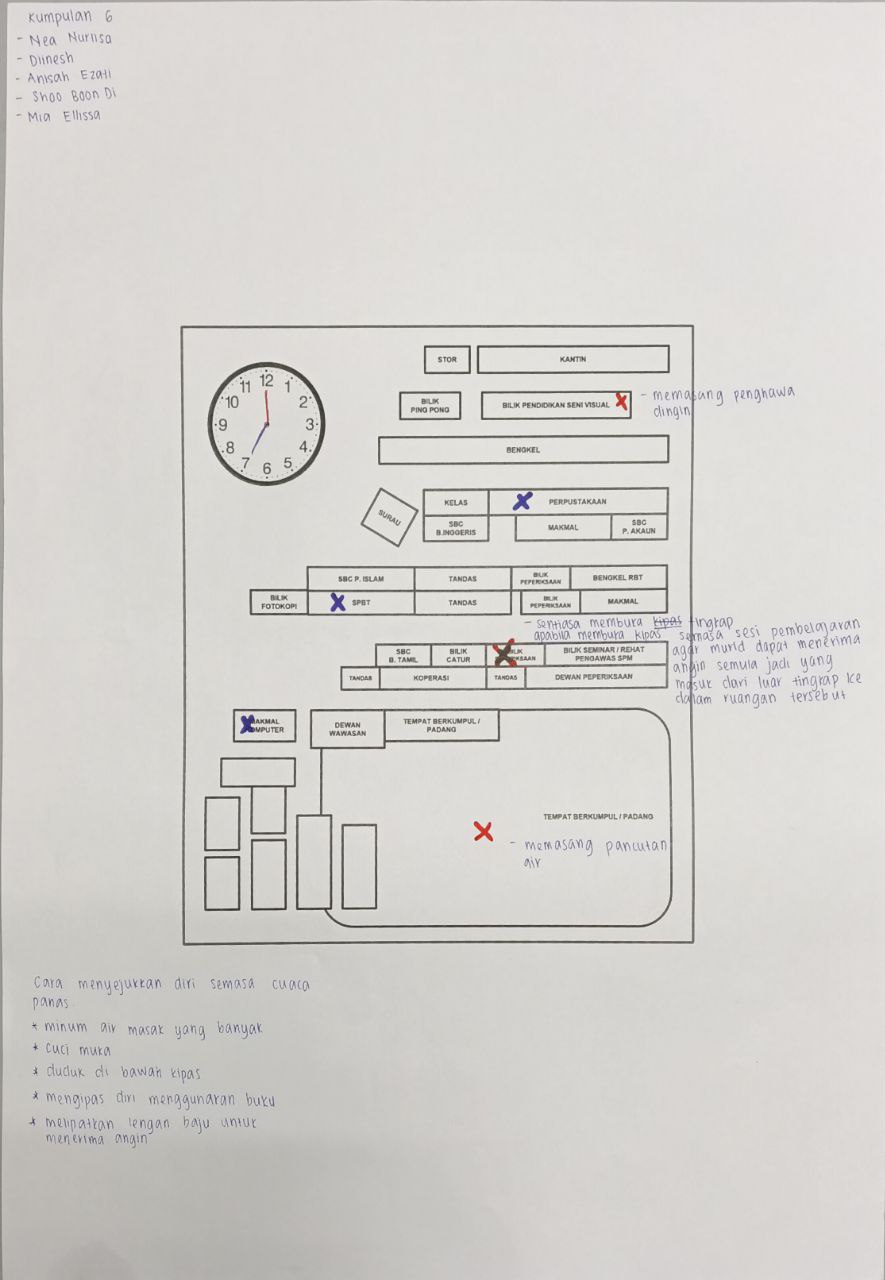


Supplementary material 8. Summary results from co-adaptation mapping and group presentation activities.

| Group | Time | | Area | | Proposed adaptation strategies | Adaptation type(s) |
| --- | --- | --- | --- | --- | --- | --- |
|  | Coldest | Hottest | Cold spot | Hot spot |  |  |
| 1 | 7.00AM | 1.30PM | Library | Workshop | Curtains, reflective roofing or insulation, additional fans or air-conditioning, regulate student density during peak heat hours. | Design, Structural, Technological |
|  |  |  | Computer lab | Prayer hall | Air-conditioning, additional vents or windows; redesign space layout for optimal airflow | Structural, Technological, Design |
|  |  |  | Teacher Office | Computer lab | Replace old and malfunctioning fans; upgrade computer systems to energy-efficient models to minimize heat output; repaint interior with soft colors to reduce psychological heat perception. | Structural, Technological, Design, Psychological |
| 2 | 6.35AM | 11.00AM | Library | Canteen | Install quieter and energy-efficient fans; close certain rooms during peak heat hours; conduct regular cleaning | Structural, Technological, Maintenance |
|  |  |  | School Textbook Room | Art room | Implement a fan operation schedule; ensure fan power remains within safe operating limits | Behavioral, Institutional |
|  |  |  | Computer lab | Islamic education room | Upgrade air conditioning system; improve ventilation layout; maintenance routines and user guidelines. | Structural, Technological, Institutional, Behavioral |
| 3 | 7.15AM | 12.30PM | Library | Classroom | Indoor vegetation (potted plants, hanging greenery); promote sitting under fans | Nature-based, Behavioral |
|  |  |  | Teacher Office | School field | Plant low-growing coconut trees; install roofed huts and shaded rest areas; encourage hydration through coconut water consumption. | Nature-based, Structural, Behavioral |
|  |  |  | Iltizam Room | Computer lab | Repair air conditioning; improve layout for even air distribution; add ceiling or standing fans; introduce safe animal enclosures. | Structural, Technological, Nature-based, Institutional |
| 4 | 7.30AM | 1.15PM | Library | Canteen | Install large, energy-efficient in high-traffic zones; add a small water fountain or misting. | Structural, Blue |
|  |  |  | Seminar room | Workshop | Introduce fans with wide rotational coverage; install air vents or wind holes; explore the use of bicycle-powered water sprinklers. | Structural, Technological, Renewable |
|  |  |  | Computer lab | School field | Plant tall shade trees along school field boundaries; eco-spaces such as small fish ponds; shaded shelters or pergolas; | Nature-based, Structural, Ecological, Green |
| 5 | 6.50AM | 1.30PM | Library | Store room | Reorganize and declutter stored materials; install additional windows. | Structural, Behavioral |
|  |  |  | Laboratory | Workshop | Install air-conditioning; ensure routine maintenance and proper placement. | Technological, Structural |
|  |  |  | Computer lab | School field | Construct shaded canopies or huts; vegetation around field perimeters; use short-sleeved uniforms and lightweight fabrics during peak heat periods | Green, Structural, Behavioral, Physiological |
| 6 | 7.00AM | 12.00PM | Library | Art room | Install energy-efficient air-conditioning; maintenance and optimal placement for effective cooling. | Technological, Structural |
|  |  |  | School Textbook Room | Examination room | Keeping windows and doors open; ceiling or standing fans. | Passive, Behavioral |
|  |  |  | Computer lab | School field | Install drinking-water stations; shaded seating zones near the field perimeter; encourage cooling practices (drink plain water, wash face, sit under fan, etc.) | Blue, Structural, Behavioral, Health-adaptive |
